# Intergenerational sustainability is enhanced by taking the perspective of future generations

**DOI:** 10.1038/s41598-021-81835-y

**Published:** 2021-01-28

**Authors:** Mostafa E. Shahen, Koji Kotani, Tatsuyoshi Saijo

**Affiliations:** 1 School of Economics and Management, Kochi University of Technology, Kochi, 780-8515 Japan; 2 Research Institute for Future Design, Kochi University of Technology, Kochi, 780-0842 Japan; 3 Faculty of Commerce, Zagazig University, Zagazig, Egypt; 4 Urban Institute, Kyusyu University, Fukuoka, Japan; 5 College of Business, Rikkyo University, Tokyo, Japan; 6 Research Institute for Humanity and Nature, Kyoto, Japan

**Keywords:** Environmental social sciences, Environmental economics, Sustainability

## Abstract

The intergenerational sustainability dilemma (ISD) is a situation of whether or not a person sacrifices herself for future sustainability. To examine the individual behaviors, one-person ISD game (ISDG) is instituted with strategy method where a queue of individuals is organized as a generational sequence. In ISDG, each individual chooses unsustainable (or sustainable) option with her payoff of $X$ ($X-D$) and an irreversible cost of $D$ (zero cost) to future generations in $36$ situations. Future ahead and back (FAB) mechanism is suggested as resolution for ISD by taking the perspective of future generation whereby each individual is first asked to take the next generation’s standpoint and request what she wants the current generation to choose, and, second, to make the actual decision from the original position. Results show that individuals choose unsustainable option as previous generations do so or $\frac{X}{D}$ is low (i.e., sustainability is endangered). However, FAB prevents individuals from choosing unsustainable option in such endangered situations. Overall, the results suggest that some new institutions, such as FAB mechanisms, which induce people to take the perspective of future generations, may be necessary to avoid intergenerational unsustainability, especially when intergenerational sustainability is highly endangered.

## Introduction

A social dilemma refers to a situation where every individual in a group or society behaves according to her self-interest without cooperating with one another, leading to a failure of maximizing the social welfare^1^. The provisions of public goods and common pool resources are considered to be intra- and inter-generational social dilemmas, and literature finds that communication enhances cooperation, leading to Pareto improvement and socially optimal outcomes^2–6^. The long-run survival of humankind on Earth is claimed to depend on whether or not we can resolve intergenerational dilemmas and maintain resources by making communication and cooperation across different generations, i.e., intergenerational sustainability (IS) problems^7–9^. However, some authors claim that it is quite challenging to make such communication and cooperation across different generations, when they are neither interacting nor overlapping^10,11^. Therefore, IS problems have occurred reflecting the lack of such communication and cooperation such as climate change, sea-level rise, accumulation of public debt and biodiversity loss^12–15^. A key question here is “does the growing threat of IS problems induce societies and individuals to take cooperative actions when communications among generations are difficult or impossible?”^16,17^. Given this state of affairs, this paper addresses how individuals cooperatively behave for maintaining IS.

We consider intergenerational sustainability dilemma (ISD) to represent a typical situation where the current generation chooses to maximize (sacrifice) her own benefits without (for) considering future generations, compromising (maintaining) IS where communications among generations cannot be made^9,18^. One of the main features in ISD is its unidirectional or irreversible nature, as the current generation affects future generations, but the opposite is not true. Thus, ISD can be considered to have a similar structure to a dictator game (DG) in which a dictator unidirectionally affects a recipient. In the unidirectional setting, the current generation (or the dictator) can prioritize her own benefits without considering future generations (or receivers). The DG has been widely studied by social scientists for the last few decades^19–26^. The stake represents the economic factor in the DG and is observed to be an influential factor in the allocations between the dictator and a receiver^27–31^. Engel^32^ reviews $$440$$ DG papers in a meta-study, identifying that the stake usually falls between 0 $ and 130 $, and an increase in the stake reduces dictators’ willingness to give. Other researchers have focused on how information on the allocations of other dictators affects a dictator’s allocation in the DG^33–38^. Ben-Ner et al.^39^ find that information about the allocations of other dictators leads a dictator to divide the allocation in a similar way to how other dictators make their allocations. In short, previous studies have shown that the economic factor and information about other dictators’ allocation influence allocations in the DG.

DG and ISD differ with respect to a distributive nature for maximizing social welfare. In ISD, sacrificing or costing oneself (for future generations) maximizes social welfare, but in DG, it is not the case. In this sense, an indirect reciprocity game can be considered the foundation of ISD, being consistent with one another in that cooperation or costing oneself for others optimizes social welfare. Yamagishi and Cook^40^ design and implement the indirect reciprocity game in a laboratory setting and study the allocation decisions between oneself and others under group generalized exchange (i.e., pay-it-forward reciprocity) and network generalized exchange treatments. In the group generalized exchange treatment, subjects’ payoffs are pooled together and each subject receives her payoff from the commonly pooled payoffs. In the network generalized exchange treatment, each subject’s payoff is determined by a subsequent subject in a loop. They find that the network generalized exchange treatment promotes cooperation, achieving higher social welfare than does the group generalized exchange treatment. Watanabe et al.^41^ experimentally examine the neural mechanisms underlying sacrificing behaviors in the pay-it-forward reciprocity game. In this game, subjects are lined up in a sequence and each subject is asked to choose between giving money to the subsequent subject and keeping it to herself. When a subject chooses giving the money to the subsequent subject, the amount of money is doubled. They find that sacrificing might be due to a desire for having emotional rewards. Horita et al.^42^ examine the effect of repeated decisions in the pay-it-forward indirect reciprocity game under a laboratory setting, finding that cooperation via self-sacrificing is transient and disappears when a subject makes the decisions repeatedly. These studies demonstrate that social welfare may not be easily optimal when people are required to sacrifice themselves.

Many scholars have applied an experimental approach in examining group behaviors regarding IS. Fischer et al.^43^ implement a common pool resource experiment with university students to investigate individual decisions in a group, demonstrating that the existence of subsequent groups motivates individuals to sustain resources. Hauser et al.^44^ conduct an online intergenerational goods experiment under a voting mechanism using a general subject pool and find that voting could reduce the exploitation of resources by restraining defectors when a majority of subjects are prosocial. Sherstyuk et al.^45^ examine the efficiency of a dynamic externality game in the laboratory, identifying that resolving the dynamic externalities becomes more challenging in intergenerational settings than in settings with infinitely lived decision makers. They also claim that access to information on the history of previous generations’ decisions may improve the negative externalities. Kamijo et al.^18^ design and implement an ISD game (ISDG) in the laboratory with a student pool to understand group behaviors in the ISD. They find that, within a group of three individuals, the introduction of an individual who is asked to play the role of deputy for future generations, called an imaginary future person, enhances IS. Shahrier et al.^9,46^ conduct an ISDG field experiment using a subject pool drawn from the general public in urban and rural areas of Bangladesh, showing that rural groups choose sustainable options more often than do urban groups, as the majority of rural people are prosocial. Moreover, they find that inducing subjects to take and understand the perspective of the next generation before making their decision, an institution called the future ahead and back mechanism, improves IS. Shahrier et al.^9,46^ note that introducing an imaginary future person in a group is not effective at maintaining IS with a general subject pool of Bangladeshi people in the ISDG field experiments. Therefore, they institute and design a future ahead and back mechanism. Overall, group behaviors in IS are mainly affected by social preferences, access to information about the decisions of previous generations (i.e., history) and institutions or environments for group decisions.

Past studies suggest that individual behaviors in the DG and group behaviors in the ISD are influenced by not only people’s social preferences of prosociality but also information about the allocations of other dictators and the decisions of previous generations, respectively. We call such information the retrospective factor for decisions in the ISD. On the other hand, how the current generation affects future generations also alters people’s behaviors in the ISD. We call this effect of the current generation’s choice on future generations the prospective factor for decisions in the ISD. This study systematically examines how individuals behave in response to the retrospective and prospective factors in the ISD and derive some implications for designing our societies to be intergenerationally sustainable. To this end, we design and institute a one-person ISD game (ISDG) with a strategy method in which a queue of individuals is organized as a generational sequence. Each individual is asked to choose either (1) an unsustainable option that yields payoff $X$, imposing an irreversible cost on future generations of $D$, or (2) a sustainable option that yields payoff $(X-D)$, without imposing any cost on future generations, in 36 situations where the histories of previous generations’ choices (the retrospective factor) and the payoff structures of $\frac{X}{D}$ (the prospective factor, i.e., the IS index) are varied. As a potential resolution of the ISD, we introduce a future ahead and back (FAB) mechanism whereby first, each individual is asked to take the position of the next generation and to request what she wants the current generation to choose and second, she makes the actual decision from the original position.

The economic factor and information about how other dictators make their allocations in the DG have been established to affect the allocations between a dictator and a receiver along with people’s social preferences. Likewise, the economic factor (i.e., $\frac{X}{D}$) and histories of previous generations’ decisions in the ISD are hypothesized to affect the allocations of the decisions made by the current generation between herself and the next generation, consequently influencing subsequent generations and IS. The ratio in ISD is interpreted to represent how many generations can enjoy the positive amount of resources before reaching the “devastating consequence” of resource extinction (i.e., $X=0$), when all the current and subsequent generations keep choosing unsustainable options. Therefore, it is very important and can be considered similar to an idea of the “tipping point” in the ecological system^8,14,47,48^. However, there is a distinction between the DG and the ISDG in that a dictator unidirectionally affects only one receiver, while the current generation unidirectionally affects not only the next generation but also all subsequent generations. To the best of our knowledge, no previous research has systematically addressed and examined individual behaviors under various situations of the ISD. Specifically, the novelties of this research lie in (1) characterizing how individuals with different social preferences behave to be sustainable or unsustainable in response to the economic (the prospective) factor and history of previous generations’ decisions (the retrospective factor) under the ISD and (2) evaluating how effective an FAB mechanism that induces people to take the standpoint of future generations is at maintaining IS.

## Results

Table 1 presents the summary statistics of experimental results for the basic one-person ISDG (basic ISDG) and the future ahead and back (FAB) treatments. The numbers of subjects who participated in the basic ISDG and FAB treatments are $55$ and $42$, among which the numbers of prosocial subjects are $30$ and $14$, respectively. Each subject went through the $36$ situations of the one-person ISDG in both treatments, generating observations of $1980$ ($= 55 \times 36$) and $1512$ ($= 42 \times 36$) in the basic ISDG and the FAB treatment, respectively. Approximately 33.7% and 44.5% of the generational choices are option $B$ in the basic ISDG and FAB treatments, implying that the percentages choosing option $A$ are 66.3% and 55.5%, respectively. These results appear to suggest that the FAB treatment is effective at inducing subjects to choose the sustainable option. To statistically confirm the difference, we run a chi-square test with the null hypothesis that the frequencies of the observations of subjects choosing options $A$ and $B$ between the basic ISDG and the FAB treatments are the same, and the null hypothesis is rejected at the 1% significance level ($\chi ^2=42.4, P<0.01$).

**Table 1:** Summery statistics.

	Basic ISDG treatment	FAB treatment
**Total no. of subjects**	55	42
No. of prosocial subjects	30 (55%)	14 (33%)
No. of proself subjects	25 (45%)	28 (67%)
No. of situations per subject	36	36
**Total number of observations**	1980	1512
Observations of choosing option $A$	1313 (66.3%)	839 (55.5%)
Observations of choosing option $B$	667 (33.7%)	673 (44.5%)

**Figure 1:**
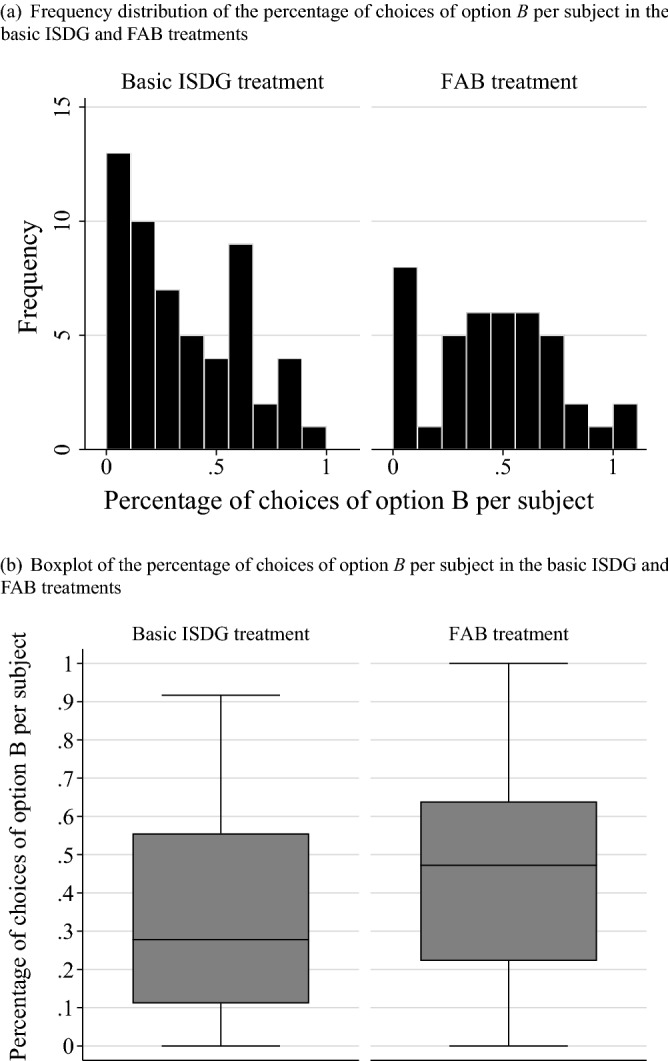
The distribution of the percentage of choices of option $B$ per subject in the basic ISDG and FAB treatments.

Figure 1a shows the frequency distributions of the percentage per subject of the choice of option $B$ in the 36 situations under the basic ISDG and FAB treatments; the percentage represents the number of situations in which the subject chooses option $B$ divided by 36 (one subject goes through 36 situations and is asked to choose between options $A$ and $B$ in each situation). Figure 1a demonstrates that the distribution under the basic ISDG treatment is skewed to the left, as the peak of the distribution is around 0–10%, indicating that a considerable portion of subjects do not choose option $B$ at all or only around 10% of the time. On the other hand, the distribution under the FAB treatment is flattened, with more concentration of around 50% as well as a reduction in the peak’s height at 0%. We also draw the corresponding boxplots in Fig. 1()for the same distributions under the basic ISDG and FAB treatments, corroborating that the location parameters, such as medians and quantiles, for the percentage of choices of option $B$ per subject in the FAB treatment are generally higher than those in the basic ISDG. We also run a Mann-Whitney test with the null hypothesis that the distributions of the percentage of choices of option $B$ per subject between the basic ISDG and FAB treatments are the same. The null hypothesis is rejected at the 10% significance level ($z=-1.79, P=0.072$), implying that subjects are more likely to choose option $B$ in the FAB treatment than in the basic ISDG treatment.

Table 2 displays the percentages of choices of option $B$ for prosocial and proself subjects in each of the basic ISDG and FAB treatments by pooling observations from subjects. The percentages of choices of option $B$ made by prosocial subjects under the basic ISDG and FAB treatments (44.72% and 55.56%) are higher than those made by proself subjects (20.44% and 38.99%). The result suggests that prosocial subjects tend to choose option $B$ more than proself subjects, which is consistent with the literature^49,50^. At the same time, the percentages of choices of option $B$ made by prosocial and proself subjects under the FAB treatments (55.56% and 38.99%) are higher than those under the basic ISDG treatment (44.72% and 20.44%). We run a chi-square test with the null hypothesis that the frequency distributions of choosing option $B$ among prosocial and proself subjects are the same between the basic ISDG and FAB treatments. The result rejects the null hypothesis at the 1% level ($\chi ^2=129.6, P<0.01$), demonstrating that the FAB treatment appears to be effective at inducing subjects to choose option $B$, irrespective of subjects’ value orientations.

**Table 2:** The percentages of option $B$ for prosocial subjects in the basic ISDG and FAB treatments.

	Percentages of option $B$ choices		
	Basic ISDG treatment	FAB treatment	Overall
Prosocial	44.72% ($\approx$ $\frac{483}{1080}$)	55.56% ($\approx$ $\frac{280}{504}$)	48.17% ($\approx$ $\frac{763}{1584}$)
Proself	20.44% ($\approx$ $\frac{184}{900}$)	38.99% ($\approx$ $\frac{393}{1008}$)	30.24% ($\approx$ $\frac{577}{1908}$)
Subtotal	33.69% ($\approx$ $\frac{667}{1980}$)	44.51% ($\approx$ $\frac{673}{1512}$)	38.37% ($\approx$ $\frac{1340}{3492}$)

To quantitatively characterize the marginal impact of subjects’ SVO and the prospective and retrospective factors on subjects’ choices in the one-person ISDG, panel logit regressions are applied to our experimental data. In the regressions, a dummy variable capturing the subject’s binary choice between options $A$ and $B$ in each situation is specified as the dependent variable, taking a choice for option $A$ as the base group. On the other hand, the SVO, the percentage of option $A$ in the sequence history, FAB treatment and the IS index ($\frac{X}{D}$) in each situation and the interaction terms of these variables are specified as the independent variables. Since one subject provides 36 observations in our experiment, the data are considered to possess a panel-data structure, where a panel unit is a subject and a time unit is one situation out of the 36. Since a time-invariant independent variable (the SVO) is included as one of the independent variables in the analysis, we apply a random-effects panel logit regression^51,52^. With these model specifications, we not only estimate the model but also calculate the marginal effect of an independent variable on the likelihood of a subject choosing option $B$^51^. Table 3 summarizes the estimation results and the associated marginal probabilities from the three panel logit regressions.

In model $1$ of Table 3, we consider the basic independent variables, consisting of the prosocial dummy, the percentage of option $A$ choices in the sequence history, the FAB treatment dummy and the IS index, finding that all the coefficients and marginal probabilities of these variables are statistically significant at 1% level. All the independent variables have a positive relationship with the probability of choosing option $B$ except the percentage of option $A$ choices in the sequence history. More specifically, subjects in the FAB treatment (prosocial subjects) are 15.8% (22.4%) more likely to choose option $B$ than those in the basic ISDG treatment (proself subjects), while an increase of one unit in the IS index leads subjects to choose option $B$ more often by 0.2%. On the other hand, subjects are 0.97% less likely to choose option $B$ as the percentage of option $A$ choices in the sequence history increases by 10%. These results indicate that prosociality and the FAB treatment are effective at maintaining IS, which is in line with previous studies on group behaviors. For example, Hauser et al.^44^ indicate that a group tends to be sustainable when a majority are prosocial individuals, while Kamijo et al., Shahrier et al. and Timilsina et al.^9,18,53^ show that the introduction of some mechanisms can have positive effects on group behaviors for IS.

In models $2$ and $3$, we include interaction terms for the FAB treatment dummy and IS index and the FAB treatment dummy and the percentage of option $A$ choices in the sequence history. The estimation results remain qualitatively the same as those in model $1$, while the interaction term of the FAB treatment dummy and IS index (FAB treatment dummy and percentage of option $A$ choices in history) is statistically significant at the 1% level (insignificant) with a negative sign in models $2$ and $3$ (in model $3$). The results suggest that subjects behave differently under the basic ISDG and FAB treatments in response to the IS index, while they do not respond to the percentage of option $A$ choices in the sequence history. Specifically, subjects tend to choose option $A$ as the IS index decreases, reflecting the result of model $1$ in Table 3. However, the results associated with the interaction terms in models $2$ and $3$ suggest that the FAB treatment prevents subjects from choosing option $A$ in response to a decrease in the IS index, making the treatment effective as sustainability becomes endangered. We apply several other models including different specifications and other interaction terms as robustness checks, yielding qualitatively similar results to those in models $1,2$ and $3$ of Table 3.

**Table 3:** Panel logit models with a dummy variable of the binary choice between options $A$ and $B$ as the dependent variable, with the choice of option $A$ is the base group.

	Model 1		Model 2		Model 3	
	Coefficients	Marginal effects$^{1}$	Coefficients	Marginal effects	Coefficients	Marginal effects
Prosocial$$^{2}$$	$$1.42^{***}$$	$$0.224^{***}$$	$$1.431^{***}$$	$$0.225^{***}$$	$$1.431^{***}$$	$$0.225^{***}$$
	(0.369)	(0.058)	(0.371)	(0.058)	(0.371)	(0.058)
% of option $$A$$ in history$$^{3}$$	$$-0.615^{***}$$	$$-0.097^{***}$$	$$-0.602^{***}$$	$$-0.095^{***}$$	$$-0.599^{***}$$	$$-0.095^{***}$$
	(0.131)	(0.021)	(0.131)	(0.021)	(0.175)	(0.021)
FAB treatment$$^{4}$$	$$1.001^{***}$$	$$0.158^{***}$$	$$1.337^{***}$$	$$0.159^{***}$$	$$1.341^{***}$$	$$0.159^{***}$$
	(0.370)	(0.059)	(0.381)	(0.058)	(0.405)	(0.058)
IS index $$\left( \frac{X}{D}\right)$$$$^{5}$$	$$0.014^{***}$$	$$0.002^{***}$$	$$0.028^{***}$$	$$0.002^{***}$$	$$0.028^{***}$$	$$0.002^{***}$$
	(0.003)	(0.0006)	(0.005)	(0.0006)	(0.005)	(0.0006)
FAB $$\times$$ IS index			$$-0.032^{***}$$	-	$$-0.032^{***}$$	-
			(0.008)	-	(0.008)	-
FAB $\times$ % of option $A$ in history					-0.007	-
					(0.265)	-
Observations	3492		3492		3492	
Wald $$\chi ^2$$	$$51.98^{***}$$		$$68.43^{***}$$		$$68.44^{***}$$	

Standard errors in parentheses.

For robustness check, we run several models with different specifications by considering other variables, such as the decision order of situations for each subject. We confirm that the main results do not change.

***,**,*Significant at the $1{\%}$, $5{\%}$ and $10{\%}$ levels, respectively.

$^{1}$Calculated at the same means of the independent variables.

$^{2}$Prosocial is a dummy variable for SVO, taking $1$ if the subject is categorized as prosocial and 0 otherwise.

$^{3}$% of choice $A$ in history is the percentage of option $A$ choices in the sequence history, taking a value from $0$ to $1$ reflecting the ratio of the number of previous generations that chose option $A$ to the total number of previous generations in the sequence history for each situation.

$^{4}$FAB treatment is a dummy variable taking $1$ if the subject is in the FAB treatment and 0 otherwise.

$^{5}$IS index is an ordered categorical variable for the ratio of $\frac{X}{D}$, taking a value from $0$ to $36.$

**Figure 2:**
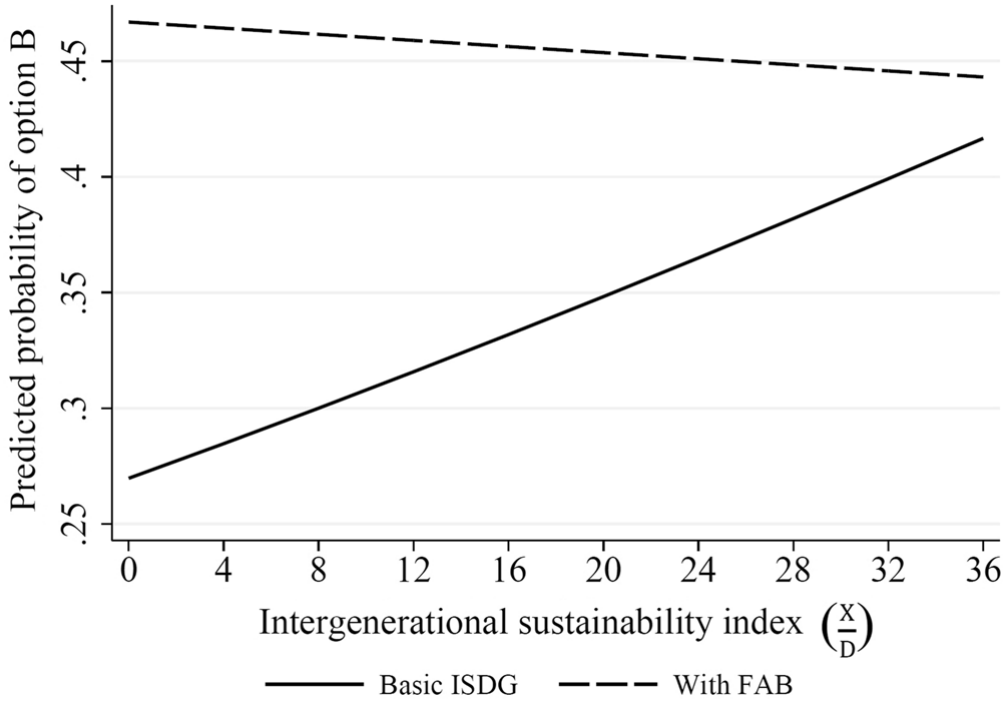
Predicted probabilities of choosing option $B$ for subjects as a function of intergenerational sustainability index in the basic ISDG and FAB treatments.

To quantitatively demonstrate how subjects behave differently under the basic ISDG and FAB treatments, we calculate the predicted probabilities of a subject choosing option $B$ over the IS index in each treatment based on the estimation result of model $2$ in Table 3. The predicted probabilities are calculated by changing the IS index, holding other independent variables fixed at the sample means. Because the interaction term of the FAB treatment dummy and IS index is estimated to be negative in model $2$, the predicted probabilities under the FAB treatment should be larger than those under the basic ISDG treatment as the IS index decreases. Figure 2 displays the predicted probabilities over the IS index under basic ISDG and FAB treatments represented by the solid and dashed lines, respectively. As seen in Fig. 2, the trajectories over the IS index are clearly different between the basic ISDG and FAB treatments. The predicted probability under the basic ISDG (solid line) increases in the IS index ranging from $0.27$ to $0.41$, while that under FAB (dashed line) is almost flat or only slightly decreases in the IS index ranging from $0.47$ to $0.44$. These results in Fig. 2 confirm that subjects tend to choose option $A$ under the basic ISDG when the IS index of a prospective factor is low. However, the introduction of the FAB can induce subjects to consistently or stably choose option $B$ irrespective of the values of the IS index.

**Figure 3:**
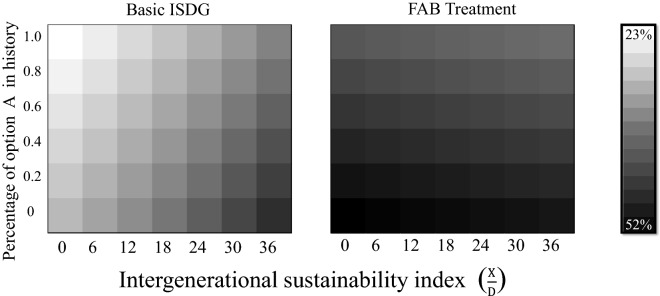
Heat map of the predicted probability of choosing sustainable option $B$ on the domain of the percentage of option $A$ choices in the sequence history and intergenerational sustainability index ($\frac{X}{D}$).

Next, we characterize how subjects respond to the retrospective and prospective factors in the ISD within a single framework. To this end, two heat maps are drawn to present the predicted probabilities of choosing option $B$ under the basic ISDG and FAB treatments on the domain of the percentage of option $A$ choices in the sequence history and the IS index (Fig. 3). The predicted probabilities are calculated based on the estimation results in model 3 of Table 3. The predicted probabilities are calculated in the same way as in Fig. 2 by holding other independent variables fixed at the sample means. In addition, as a robustness check, they are calculated based on the estimation results in model 2. We confirm that they remain qualitatively the same as in Fig. 3. The vertical (horizontal) axis represents the percentage of option $A$ choice in the sequence history (IS index), and it varies from $0$ to $1$ (from $0$ to $36$). The density of the black color in each location of the domain reflects the predicted probability of choosing option $B$; the darker the color, the higher is the predicted probability. The scale, ranging from 23 to 52%, is shown on the right-hand side in Fig. 3.

The predicted probabilities under the basic ISDG in Fig. 3 corroborate that subjects are more likely to choose option $A$ as the IS index (the percentage of option $A$ in history) becomes lower (higher), consistent with the results in Table 3 and Fig. 2. This is quite intuitive in the sense that people in the current generation tend to give up being sustainable when previous generations chose such unsustainable options that it may be too late or the situation faced by the current generation too grave for sustainability to be improved. However, the predicted probabilities under the FAB treatment in Fig. 3 show that subjects tend to choose option $B$ stably and consistently, being more invariant against changes in either the IS index or the percentage of option $A$ in history than the probabilities in the basic ISDG. In fact, the predicted probabilities under the FAB treatment range from 40 to 52%, demonstrating that asking subjects to take the position of the next generation fundamentally affects their choices between options $A$ and $B$ in response to the retrospective and prospective factors in the ISD. Overall, the regression results in Table 3, Figs. 2 and 3 establish that people react to the retrospective and prospective factors in an intuitive way under the basic ISDG, implying that people in the current generation choose unsustainability if previous generations betray them and it seems too late for the current situation to be made sustainable. However, the FAB treatment is demonstrated to prevent people from making such choices.

## Discussion

Some behavioral scientists and economists have recently emphasized the importance of analyzing economic, cognitive and noncognitive factors to characterize human behaviors at the individual and group levels in a single framework^54–58^. Our experiments are considered to systematically examine individual behaviors in response to these factors under the ISD in the sense that prospective and retrospective factors and social preferences are known to correspond to economic and noncognitive factors, respectively^54^. Overall, the results are interpreted to demonstrate that the economic factors of the IS index and the percentage of option $A$ choices in the sequence history as well as social preferences have impacts on individual behaviors in the ISD in an intuitive way, consistent with the literature on the dictator and other games. In particular, social preference of prosociality is identified as one influential factor in subjects choosing the sustainable option in the ISDG, and a similar result is consistently confirmed in common pool resource and public goods games^9,18,44,59^. However, people’s social preferences are claimed to be determined at young ages by the culture and social norms of societies, remaining fixed when they become adults. Therefore, these preferences are considered impossible to change with policy or external interventions^60–62^.

This paper finds that FAB mechanism is an effective treatment to prevent individuals from choosing an unsustainable option, even when intergenerational sustainability is endangered. It is our belief that uniqueness of FAB mechanism lies in a whole package of (1) explicitly asking individuals to take the next generation’s perspective and making a request to the previous generation and (2) to make the actual decision after going back to the original position. In other words, such explicit procedures in FAB mechanism induce individuals to have a direct and specific image of future generations’ emotions and feelings. An important question here is why and how the FAB mechanism affects individual behaviors in the ISD. Although we admit that there are several possible explanations, we conjecture that the FAB mechanism affects a cognitive factor in human-decision processes^63^. In particular, Cooper^64^ argues that some dissonance in human cognition, that is, cognitive dissonance, may influence human decisions when individuals experience two or more different psychological and/or economic representations in a decision-making situation, such as a social dilemma, where two representations conflict with one another regarding interests and payoffs. Since the FAB mechanism requires each individual to experience or role-play two representations of the current and future generations where each generation’s interest conflicts, we argue that cognitive dissonance in subjects’ decision-making processes might have been triggered and augmented to enhance sustainable choices over the outcomes observed in the basic ISDG.

Another possible explanation is that the FAB mechanism might affect not only cognitive factors but also noncognitive factors in human decision-making processes. Some economists, psychologists and neuroscientists demonstrate that empathy is a primary factor in characterizing prosocial behaviors in several different games and settings and is known to play a part in cognitive and noncognitive factors^65–70^. In economics, Andreoni and Rao^71^ and Andreoni et al.^72^ demonstrate that prosocial donations are increased in the DG by letting one subject role-play both the dictator and the receiver. They argue that empathy from the dictator to the receiver is enhanced by such role-playing and is a key means of promoting prosocial behaviors. Furthermore, psychologists argue that empathy can be a main factor in making decisions to the benefit of others or engaging in prosocial behaviors even at a personal cost^65^. In the ISDG, choosing the sustainable option is equivalent to benefiting others at a personal cost. Thus, the FAB mechanism may be considered to enhance the empathy of the current generation through its role-playing of the next generation in the ISD.

Democracy and capitalism have become two major social institutions that have been adopted by many countries in the world over the last few decades. However, some social scientists argue that these institutions are not future-oriented but present-oriented in their nature^73,74^. Democracy and capitalism rarely require people to take the standpoint of future generations, even for intergenerational problems such as climate change and government debt, thus the decisions end up being mostly made from the current generation’s standpoint^8,13,14,23,75–77^. Corporations and private companies, as integral parts of capitalism, sometimes follow the same practices leading to undermining corporate sustainability, i.e., environmental integrity, social equity and economic prosperity^78,79^. Our findings imply that IS problems will worsen in the absence of a new mechanism to affect people’s cognitive and/or noncognitive processes. They also suggest that the FAB mechanism is one approach to nudge the current generation toward being future-oriented. We believe that institutionalization of the FAB mechanism is one possible resolution for the ISD, affecting people’s cognitive and noncognitive factors by propagating an idea of “putting oneself in future generations’ shoes”, and it shall be effective at the individual, organizational and societal levels. Therefore, this simple FAB treatment can be implemented as a mechanism by corporations and public sectors to expand the way in which employees, stakeholders and general public think about environment, long-term investment opportunities and new products or services as key components of sustainability through including future generations’ perspectives.

Finally, we note some limitations and future avenues of research. Our research does not address the detailed processes and channels of how and why the FAB mechanism affects individual behaviors in the ISD. To address these issues, two approaches can be suggested: (1) a neuropsychological approach and (2) qualitative and deliberative interviews. The neuropsychological approach should allow the collection of various psychological scales and neuroimages to examine the possible processes and channels engaged when individuals make decisions under the FAB mechanism in the ISDG. In this way, a specific factor that influences individual behaviors may be identified^80,81^. Qualitative interviews and deliberative approaches have already been used by some economists and psychologists^82–86^. Individual interviews or group deliberations are conducted to clarify how individuals and groups reach decisions^87^. Specifically, qualitative content analyses and text mining can be applied to untangle the detailed changes in individual behaviors that occur under the FAB mechanism in the ISDG. These caveats notwithstanding, it is our belief that this paper is an important first step in understanding individual behaviors in the ISD and suggests a possible mechanism to enhance sustainability.

## Methods

We administered a one-person intergenerational sustainability dilemma game (ISDG), social value orientation (SVO) game and questionnaires to collect data on individual behaviors, social preferences and sociodemographic information from subjects. This study was approved by the research ethics committee of Kochi University of Technology. The methods were carried out in accordance with the approved guidelines and regulations. Subjects provided their written informed consent to participate in this study.

### One-person intergenerational sustainability dilemma game (One-person ISDG)

We designed and implemented a one-person ISDG, which possesses similar structures to those of the ISDG played by a group of three people in Kamijo et al.^18^ and Shahrier et al.^9^. A one-person ISDG is organized by queuing a sequence of consecutive generations, and each generation is represented by one person. A generation is asked to make a choice between an unsustainable option $A$ and a sustainable option $B$. If a generation chooses option $A$, she receives a payoff of $X$ tokens (hereafter, we skip mentioning “tokens”), and the next generation faces the decision environment where the payoffs associated with options $A$ and $B$ uniformly decrease by $D$. If a generation chooses option $B$, she receives a payoff of $X-D$, and the next generation has the same decision environment as the current one, where the payoffs associated with options $A$ and $B$ never decrease. An essential feature of the game is that the current generation affects subsequent generations, while the opposite is not true. The 1st generation always starts a one-person ISDG with option $A=3600$ and option $B=3600-D$ in any situation. Suppose that a subject is the 1st generation and plays the game with $D=900$ in a specific situation. The 1st generation receives $3600$ if she chooses option $A$, and the 2nd generation plays the game with options $A=2700$ and $B=1800$. When the 1st generation chooses option $B$, she receives $2700$ and the 2nd generation plays the game with options $A=3600$ and $B=2700$.

A strategy method is applied to create $36$ different one-person ISDG situations that each subject goes through^88^. Specifically, the strategy method applied in this research follows a conditional information lottery (CIL) method^89,90^. The CIL method enables us to create some fictional situations and one real situation where subjects can not distinguish between the fictional ones and real one. The $36$ situations in this experiment consist of $35$ fictional situations, which are uniformly applied for all the subjects, and one real situation (i.e. binding situation), which is different for each subject. Refer to the “supplementary information” for the detailed explanations as well as the ways regarding how we create the 36 situations in ISDG by parametrization of (1) the decisions of previous generations as a retrospective factor and (2) $\frac{X}{D}$ as a prospective factor. We call a series of the benchmark experimental procedures in which each subject plays the 36 situations “basic ISDG treatment”.

Building upon the basic ISDG treatment, we apply the future ahead and back (FAB) mechanism for the one-person ISDG in 36 situations, which is hereafter called the “FAB treatment”. In the FAB treatment, we ask each subject to go through the following steps in each situation. As the 1st step, each subject is asked to imagine that she is in the next generation. From the standpoint of the next generation, she is asked to make a request about the choice that she wants the previous generation to choose between options $A$ and $B$. As the 2nd step, the subject is asked to return to her original (actual) position in the sequence, and she makes her final and actual decision by choosing one option, $A$ or $B$, for that situation. For instance, if a subject is the 5th generation in the sequence for one situation, then she is asked to imagine herself in the position of the 6th generation in the sequence and to make a request about the choice that she wants the 5th generation in the sequence to make. After that, she is asked to return to her original position in the sequence (i.e., the 5th generation) and make her final and actual choice for that situation. Each subject was randomly assigned to either the basic ISDG treatment or the FAB treatment and played the one-person ISDG with a strategy method in 36 different situations, consisting of the 35 fictional situations and a single binding situation. The orders of the 36 situations that each subject went through in the one-person ISDG were randomly shuffled to avoid order effects. In the one-person ISDG, one experimental token was calculated and exchanged as $1.5$ JPY, and subjects were paid $3000$ JPY ($\approx 27.8$ USD) on average.

#### Social value orientation

Subjects’ social preferences are proxied by their social value orientations (SVOs), which were identified using the triple dominance measure^91^. This measure consists of 9 items, each of which contains three choices. For each item, subjects must make one choice over how to divide an amount of money between herself and a stranger. For example, each subject faces the following three options: $A$: you get $500$ and the other gets $100$, $B$: you get $500$ and the other gets $500$ and $C$: you get $560$ and the other gets $330$. A competitive subject is likely to choose option $A$, maximizing the gap between her own and the stranger’s points $(500-100=400)$. A prosocial subject has high chances of choosing option $B$, as it maximizes the joint benefit $(500+500=1000)$. An individualistic subject chooses option $C$ by maximizing her payoff without considering the other^92^. A subject’s type, i.e., individualistic, competitive or prosocial, is identified by her choices in the SVO game. When a subject makes $6$ consistent choice for the same orientation (i.e., individualistic, competitive or prosocial) out of the 9 items, then she is considered to have that orientation or otherwise is “unidentified”. Subjects were randomly paired for the computation of their payoffs based on their performance, and they were paid on average $500$ JPY ($\approx 4.7$ USD) in the SVO game.

#### Experimental procedures

Our experiments were conducted at experimental laboratories at Kochi University of Technology. The experiment comprised 27 sessions, each involving $4\sim 5$ subjects, for a total of $104$ subjects ($55$ females and $49$ males; $\text {average age}=20.4$). The observations of 6 subjects in the FAB treatment and 1 subjects in the basic ISDG treatment were dropped because of missing responses in the one-person ISDG, which made the number of subjects in the FAB treatment lower than that in the basic ISDG treatment. The subjects were volunteer undergraduate students in various fields such as engineering and social science; each subject participated in only one session and was paid in total $4000$ JPY ($\approx 37$ USD) on average. The time of each session varied between the basic ISDG and FAB treatments. One session in the basic ISDG treatment consisted of two parts and took approximately $75$ min. In the first part, subjects completed the one-person ISDG for $40$ min. In the second part, they completed the SVO game and questionnaires for $35$ min. One session in the FAB treatment also consisted of two parts and took approximately $90$ min. In the first part, subjects completed the one-person ISDG for $55$ min—a longer duration than that of the basic ISDG treatment due to the additional procedures in the FAB (see the 1st and 2nd steps of the FAB treatment within the dashed-line box at Fig. S2 in the supplementary information). In the second part, they complete the SVO game and questionnaires for $35$ min.

Upon arriving to the meeting room, each subject picked a lottery number that determined her experimental ID. Then, the subjects were taken to two different designated rooms based on their experimental IDs. In the basic ISDG treatment, each subject read the experimental instructions and listened to an oral presentation made by an experimenter about the basic one-person ISDG. We use neutral terminologies in the explanations and avoid using terms such as “generations”, “sustainable” and “unsustainable”. Then, each subject completed the $36$ situations of the basic one-person ISDG treatment in a shuffled order. Each subject made her decision by choosing between options $A$ and $B$ in each of the situations. When a subject finished making the decisions in all 36 situations, she was informed of the situation number that corresponded to the binding situation, which determined her final payoff from the one-person ISDG. Then, subjects moved to a different room to complete the SVO game and fill out the questionnaires. After that, the subjects moved to a payment room, where the payment for the SVO game was calculated by randomly pairing subjects together. In the FAB treatment, each subject follow the same steps of basic ISDG treatment in addition to a perspective-taking step as follows. In each situation, the subject was asked to imagine that she was in the position of the next generation in the sequence. From that position, she made a request to the previous generation on which choice she wanted the previous generation to make. After that, she returned to her original position in the sequence and made her final decision between options $A$ and $B$. A flow chart is presented in the supplementary information to show the procedures for the one-person ISDG, SVO game and questionnaire in one session for the basic ISDG and FAB treatments.

## Supplementary Information

Supplementary material 1

Supplementary material 2

Supplementary material 3

Supplementary material 4
