# Supplementary material for: Intergenerational sustainability is enhanced by taking the perspective of future generations
Source: Sci Rep. 2021 Jan 28;11:2437. doi: 10.1038/s41598-021-81835-y (PMC7844004; doi:10.1038/s41598-021-81835-y)

Supplementary information: The detailed experimental procedures and methods for the manuscript "Intergenerational sustainability is enhanced by taking the perspective of future generations"

Mostafa E. Shahen[*,†] Koji Kotani[*,†,‡,§,¶] Tatsuyoshi Saijo[*,†,‡,‖]

December 19, 2020

[*]Research Institute for Future Design, Kochi University of Technology
[†]School of Economics and Management, Kochi University of Technology
[‡]Urban Institute, Kyusyu University
[§]College of Business, Rikkyo University
[¶]Corresponding author, e-mail: kojikotani757@gmail.com
[‖]Research Institute for Humanity and Nature

1 Detailed experimental method

We administered a one-person intergenerational sustainability dilemma game (ISDG), social value orientation (SVO) game and questionnaires to collect data on individual behaviors, social preferences and sociodemographic information from subjects.

One-person intergenerational sustainability dilemma game (One-person ISDG)

We designed and implemented a one-person ISDG, which possesses similar structures to those of the ISDG played by a group of three people in Kamijo et al. (2017) and Shahrier et al. (2017). A one-person ISDG is organized by queuing a sequence of consecutive generations, and each generation is represented by one person. A generation is asked to make a choice between an unsustainable option A and a sustainable option B. If a generation chooses option A, she receives a payoff of X tokens (hereafter, we skip mentioning "tokens"), and the next generation faces the decision environment where the payoffs associated with options A and B uniformly decrease by D. If a generation chooses option B, she receives a payoff of $X - D$, and the next generation has the same decision environment as the current one, where the payoffs associated with options A and B never decrease. An essential feature of the game is that the current generation affects subsequent generations, while the opposite is not true.

The 1st generation always starts a one-person ISDG with option $A = 3600$ and option $B = 3600 - D$ in any situation. Suppose that a subject is the 1st generation and plays the game with $D = 900$ in a specific situation. The 1st generation receives 3600 if she chooses option A, and the 2nd generation plays the game with options $A = 2700$ and $B = 1800$. When the 1st generation chooses option B, she receives 2700 and the 2nd generation plays the game with options $A = 3600$ and $B = 2700$. Next, suppose that a subject is the 5th generation and plays the game with $D = 300$ in another situation, given a history that the 1st and 3rd (2nd and 4th) generations chose option A (B). In this case, the 5th generation faces the decision environment where the payoffs associated with options A and B are 3000 ($= 3600 - 2D = 3600 - 2 \times 300$) and 2700, respectively, noting that the two previous generations choose option A. Therefore, the 5th generation receives 3000 if she

chooses option A, and the 6th generation plays the game with options $A = 2700$ and $B = 2400$. If the 5th generation chooses option B, she receives 2700, and the 6th generation plays the game with options $A = 3000$ and $B = 2700$.

[Table 1 about here.]

A strategy method is applied to create 36 different one-person ISDG situations that each subject goes through (Selten, 1967). Specifically, the strategy method applied in this research follows a conditional information lottery (CIL) method (Bardsley, 2000, Bardsley and Sausgruber, 2005). The CIL method enables us to create some fictional situations and one real situation where subjects can not distinguish between the fictional ones and real one. The 36 situations in this experiment consist of 35 fictional situations, which are uniformly applied for all the subjects, and one real situation (i.e. binding situation), which is different for each subject. In the 35 situations, the history of previous generations' choices, the payoff of X that a generation can receive, a payoff difference of D between options A and B and the ratio between X and D (i.e., $\frac{X}{D}$) are parametrized under the assumptions that the 1st generation always starts the one-person ISDG with options $A = 3600$ and $B = 3600 - D$ and that the value of D remains the same in each situation. Table 1 summarizes the 35 different situations in the one-person ISDG, listing the associated percentages of previous generations that choose unsustainable option A in history, ranging from 0 to 1; the payoff X that a generation can receive, ranging from 0 to 3600; the difference D, ranging from 100 to 1800; and the ratio between X and D, ranging from 0 to 36. Although table 1 contains the percentage of previous generations in history for each situation that chose option A as a summary, a subject is shown a whole history of how each previous generation chose between options A or B, displayed by a sequence of human-shaped icons with different colors in each situation as shown in tables 2 and 3.

[Table 2 about here.]

[Table 3 about here.]

Figure 1 displays a scatter plot for the distribution of the 35 situations over the percentage of previous generations who choose option A and the ratio between X and D, where each plot corresponds to one situation in table 1. In this experimental design, the history of the sequence for each situation and the ratio between X and D for each situation can be interpreted as the retrospective and prospective factors because they represent what happened in the past as well as what will happen to the subsequent generations in the sequence for each situation, respectively. Specifically, the history of the sequence for each situation is interpreted as the retrospective factor, while the ratio of $\frac{X}{D}$ is interpreted as the prospective factor, representing how many generations in the sequence can receive a positive payoff of X for each situation when each generation keeps choosing option A. We call the ratio of $\frac{X}{D}$ the intergenerational sustainability index (i.e., IS index) in the one-person ISDG. The parametrization is made to widely vary the retrospective (history) and prospective ($\frac{X}{D}$) factors as well as to minimize the correlation among the factors in the one-person ISDG with a strategy method, reflecting figure 1 ($r = 0.099, P = 0.56$). For example, the 23rd situation in table 1 consists of a history in which $70\,\%$ of previous generations chose option A, $X = 1500$ and $D = 300$, implying that the current generation is 11th and there are 10 previous generations. Concretely, the history consists of 7 previous generations (i.e., 1st, 2nd, 4th, 6th, 8th, 9th and 10th) that chose option A and of 3 previous generations (i.e., 3rd, 5th, 7th) that chose option B, as shown in figure 2. In this case, the payoffs associated with options A and B that the 11th generation faces are 1500 ($= 3600 - 7D = 3600 - 7 \times 300$) and 1200, respectively.

[Figure 1 about here.]

Figure 2 shows the screens of the game, which are designed following Strombach et al. (2015). In each situation, a subject observes the screen of the game when she is asked to decide between options A and B. Here, we take the 23rd situation as an example. The first screen in figure 2 notifies the subject of the situation number (i.e., the 23rd situation), and the second screen presents the history, options and associated payoffs for the current and next generations. At the top of the second screen, human-shaped icons represent the generations in each situation, and the dotted and striped icons represent the current and subsequent generations, respectively. The gray and light

gray icons represent the previous generations in history who chose options A and B, respectively, while the black icons represent the subsequent generations to come after the next generation. In the middle of the screen, the options for the current and next generations are presented next to the white and striped icons, respectively.

[Figure 2 about here.]

In addition to these 35 situations of the one-person ISDG, each subject plays one binding situation whose decision environments evolve over generations according to how previous generations have chosen and how the current generation chooses, being passed to the subsequent generations within the sequence to determine the real payment to subjects. In the binding situation, the 1st generation starts the game with option $A = 3600$, where one value of D is randomly picked from the four possible values of 300, 600, 900 and 1200. Once it is picked, the value of D remains the same for the $1\text{st}, 2\text{nd}, \dots$ generations in the sequence for the binding situation. The binding situation is continued as long as the value of X is strictly positive and ends when it becomes zero or negative for some generation in the sequence. Therefore, the payoff structures in the decision environment faced by each generation in the sequence for the binding situation are different, while the 35 situations in table 1 are uniformly played by all subjects. We call a series of the benchmark experimental procedures in which each subject plays the 36 situations "basic ISDG treatment."

Building upon the basic ISDG treatment, we apply the future ahead and back (FAB) mechanism for the one-person ISDG in 36 situations, which is hereafter called the "FAB treatment." In the FAB treatment, we ask each subject to go through the following steps in each situation. As the 1st step, each subject is asked to imagine that she is in the next generation. From the standpoint of the next generation, she is asked to make a request about the choice that she wants the previous generation to choose between options A and B. As the 2nd step, the subject is asked to return to her original (actual) position in the sequence, and she makes her final and actual decision by choosing one option, A or B, for that situation. For instance, if a subject is the 5th generation in the sequence for one situation, then she is asked to imagine herself in the position of the 6th generation in the sequence and to make a request about the choice that she wants the 5th generation in the sequence

to make. After that, she is asked to return to her original position in the sequence (i.e., the 5th generation) and make her final and actual choice for that situation.

Each subject was randomly assigned to either the basic ISDG treatment or the FAB treatment and played the one-person ISDG with a strategy method in 36 different situations, consisting of the 35 fictional situations and a single binding situation. The orders of the 36 situations that each subject went through in the one-person ISDG were randomly shuffled to avoid order effects. The experimenters offered the following explanation to the subjects: "One situation out of the 36 situations shall be chosen for the actual experimental payment, following a certain rule. Because you do not know in advance which situation shall be chosen for the payment, please be serious and considerate about a choice in each situation that may affect the subsequent subjects, because they will play after you." However, in reality, to simplify the experimental procedures, the experimenters predetermined that the choices and outcomes in the binding situation would only be used to determine the experimental payment of each subject and to affect the subsequent subjects. In the one-person ISDG, one experimental token was calculated and exchanged as 1.5 JPY, and subjects were paid 3000 JPY (≈ 27.8 USD) on average.

2 Experimental procedures

Our experiments were conducted at experimental laboratories at Kochi University of Technology. The experiment comprised 27 sessions, each involving $4 \sim 5$ subjects, for a total of 104 subjects (55 females and 49 males; average age $= 20.4$). The observations of 6 subjects in the FAB treatment and 1 subjects in the basic ISDG treatment were dropped because of missing responses in the one-person ISDG, which made the number of subjects in the FAB treatment lower than that in the basic ISDG treatment. The subjects were volunteer undergraduate students in various fields such as engineering and social science; each subject participated in only one session and was paid in total 4000 JPY ($\approx$ 37 USD) on average. The time of each session varied between the basic ISDG and FAB treatments. One session in the basic ISDG treatment consisted of two parts and

took approximately 75 minutes. In the first part, subjects completed the one-person ISDG for 40 minutes. In the second part, they completed the SVO game and questionnaires for 35 minutes. One session in the FAB treatment also consisted of two parts and took approximately 90 minutes. In the first part, subjects completed the one-person ISDG for 55 minutes—a longer duration than that of the basic ISDG treatment due to the additional procedures in the FAB (see the 1st and 2nd steps of the FAB treatment within the dashed-line box in figure 3). In the second part, they complete the SVO game and questionnaires for 35 minutes.

Figure 3 presents a flow chart for the procedures of the one-person ISDG, SVO game and questionnaire in one session for the basic ISDG and FAB treatments. Upon arriving to the meeting room, each subject picked a lottery number that determined her experimental ID. Then, the subjects were taken to two different designated rooms based on their experimental IDs. In the basic ISDG treatment, each subject read the experimental instructions and listened to an oral presentation made by an experimenter about the basic one-person ISDG. We use neutral terminologies in the explanations and avoid using terms such as "generations," "sustainable" and "unsustainable." Then, each subject completed the 36 situations of the basic one-person ISDG treatment in a shuffled order. Each subject made her decision by choosing between options A and B in each of the situations. When a subject finished making the decisions in all 36 situations, she was informed of the situation number that corresponded to the binding situation, which determined her final payoff from the one-person ISDG. Then, subjects moved to a different room to complete the SVO game and fill out the questionnaires. After that, the subjects moved to a payment room, where the payment for the SVO game was calculated by randomly pairing subjects together. In the FAB treatment, each subject follow the same steps of basic ISDG treatment in addition to a perspective-taking step as follows. In each situation, the subject was asked to imagine that she was in the position of the next generation in the sequence. From that position, she made a request to the previous generation on which choice she wanted the previous generation to make. After that, she returned to her original position in the sequence and made her final decision between options A and B.

[Figure 3 about here.]

Screen of one-person ISDG game

Figure 4(a) shows the screens that a subject observes while playing the basic ISDG and FAB treatments. The screens for the basic ISDG treatment are displayed and two screens presented in each situation. The first screen presents the situation number and appears for 3 seconds. After that, the second screen appears for 15 seconds and presents the history of the previous generations' choices at the top of the screen and the options available for the current and subsequent generations in the middle. We call the second screen the "one-person ISDG screen." During the time in which the second screen is displayed, each subject makes her decision by entering the character "A" or "B" in another computer display served as a response device. A subject has to go through the above processes by observing the first and second screens in each situation, and the one-person ISDG is continued until she finishes making the decisions in all 36 situations.

Figure 4(b) presents a series of screens that a subject faces for each situation under the FAB treatment in the one-person ISDG. The first screen presents the situation number for 3 seconds. The second screen is the same screen as the second screen in the basic ISDG treatment (i.e., the one-person ISDG screen), which is displayed for 4 seconds to familiarize subjects with the decision environment. The third screen is displayed to notify the subject that she should imagine herself in the position of the next generation in the sequence and make a request about which choice she wants the previous generation to make between options A and B. Then, the one-person ISDG screen is displayed again for 10 seconds. At that time, the subject must make a request of the previous generation by entering the character "A" or "B" in another computer display served as a response device. After that, another notice screen appears for 3 seconds to let the subject know that she must return to her original position. The one-person ISDG screen appears one more time for 10 seconds to present the one-person ISDG choices to the subject, and she makes her final choice from her original position in the current generation. Subjects make their final choice by entering "A" or "B" in the response device, while the request they have made as the next generation kept visible on the display of the response devise. As in the basic ISDG treatment, a subject has to go through the above processes by observing a series of screens in each situation, and the one-person

ISDG is continued until she finishes making the decisions in all 36 situations.

[Figure 4 about here.]

References

Bardsley, N. (2000). Control without deception: Individual behaviour in free-riding experiments revisited. *Experimental economics*, 3:215–240.

Bardsley, N. and Sausgruber, R. (2005). Conformity and reciprocity in public good provision. *Journal of economic psychology*, 26:664–681.

Kamijo, Y., Komiya, A., Mifune, N., and Saijo, T. (2017). Negotiating with the future: Incorporating imaginary future generations into negotiations. *Sustainability science*, 12:409–420.

Selten, R. (1967). Die strategiemethode zur erforschung des eingeschränkt rationalen verhaltens imrahmen eines oligopolexperiments. In Sauermann, H., editor, *Beiträge zur Experimentellen Wirtschaftsforschung*, pages 136–168. Tübingen: JCB Mohr (Paul Siebeck).

Shahrier, S., Kotani, K., and Saijo, T. (2017). Intergenerational sustainability dilemma and the degree of capitalism in societies: A field experiment. *Sustainability science*, 12:957–967.

Strombach, T., Weber, B., Hangebrauk, Z., Kenning, P., Karipidis, I., Tobler, P., and Kalenscher, T. (2015). Social discounting involves modulation of neural value signals by temporoparietal junction. *Proceedings of the National Academy of Sciences of the United States of America*, 112:1619–1624.

List of Tables

1 The 35 situations that each subject plays in one-person ISDG 12
2 Detailed descriptions with human-shaped icon displays in history from 1 to 21 situations within the 35 ones as seen by each subject 13
3 Detailed descriptions with human-shaped icon displays in history from 22 to 35 situations within the 35 ones as seen by each subject (continuum from where we left off in table 2) 14

Table 1: The 35 situations that each subject plays in one-person ISDG

Situations	% of option A in history	X	D	$\frac{X}{D}$	# of generations in history	Current generation		
						Position[1]	Option A	Option B
1	0	3600	1800	2	0	1	3600	1800
2	0	3600	1200	3	5	6	3600	2400
3	0	3600	900	4	7	8	3600	2700
4	0	3600	300	12	0	1	3300	3300
5	0	3600	100	36	9	10	3600	3500
6	0.25	2700	900	3	4	5	2700	1800
7	0.25	1800	300	6	8	9	1800	1500
8	0.25	3400	200	17	4	5	3400	3200
9	0.33	0	1200	0	9	10	0	-1200
10	0.33	1200	600	2	12	13	1200	600
11	0.5	0	1800	0	4	5	0	-1800
12	0.5	0	900	0	8	9	0	-900
13	0.5	1200	1200	1	4	5	1200	0
14	0.5	2400	600	4	4	5	2400	1800
15	0.5	2400	600	4	4	5	2400	1800
16	0.5	2400	300	8	8	9	2400	2100
17	0.5	3400	200	17	2	3	3400	3200
18	0.5	3200	100	32	8	9	3200	3100
19	0.63	2600	200	13	8	9	2600	2400
20	0.67	1200	1200	1	3	4	1200	0
21	0.67	3000	300	10	3	4	3000	2700
22	0.67	2600	100	26	15	16	2600	2500
23	0.7	1500	300	5	10	11	1500	1200
24	0.7	2200	100	22	20	21	2200	2100
25	0.75	0	300	0	16	17	0	-300
26	0.75	900	900	1	4	5	900	0
27	0.75	1800	600	3	4	5	1800	1200
28	0.75	3300	100	33	4	5	3300	3200
29	0.78	0	200	0	23	24	0	-200
30	1	1800	1800	1	1	2	1800	0
31	1	1800	900	2	2	3	1800	900
32	1	2400	1200	2	1	2	2400	1200
33	1	3300	300	11	1	2	3300	3000
34	1	3000	200	15	3	4	3000	2800
35	1	3500	100	35	1	2	3500	3400

[1] This represents current generation position in a situation. For example, in situation number 23, the number of generations in history is 10, thus current generation position is the 11th generation.

Table 2: Detailed descriptions with human-shaped icon displays in history from 1 to 21 situations within the 35 ones as seen by each subject

Situations	Human-shaped icons in history	% of option A in history	X	D	X/D
1	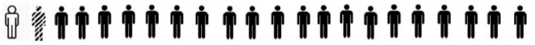	0	3600	1800	2
2	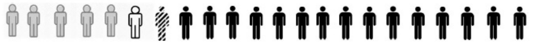	0	3600	1200	3
3	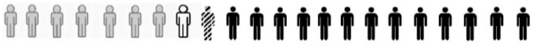	0	3600	900	4
4	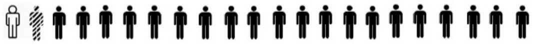	0	3600	300	12
5	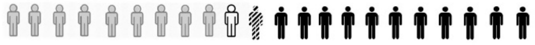	0	3600	100	36
6	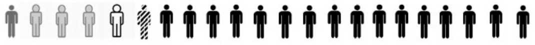	0.25	2700	900	3
7	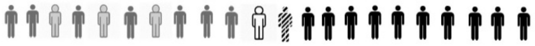	0.25	1800	300	6
8	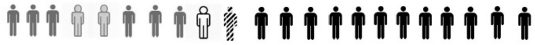	0.25	3400	200	17
9	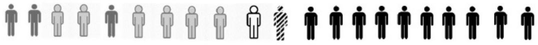	0.33	0	1200	0
10	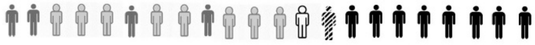	0.33	1200	600	2
11	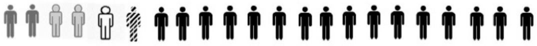	0.50	0	1800	0
12	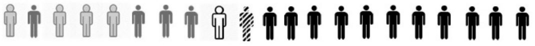	0.50	0	900	0
13	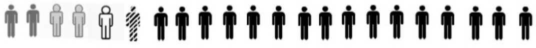	0.50	1200	1200	1
14	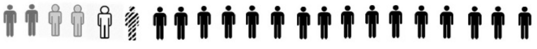	0.50	2400	600	4
15	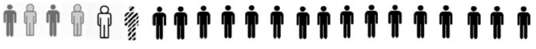	0.50	2400	600	4
16	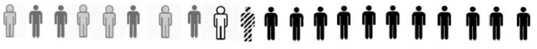	0.50	2400	300	8
17	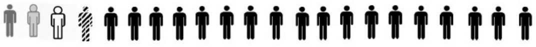	0.50	3400	200	17
18	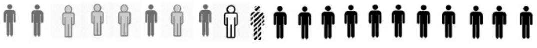	0.50	3200	100	32
19	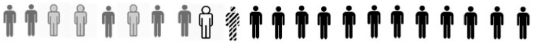	0.63	2600	200	13
20	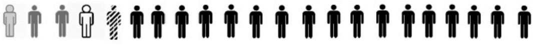	0.67	1200	1200	1
21	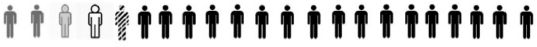	0.67	3000	300	10

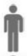 Pervious generations who chose option A

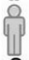 Pervious generations who chose option B

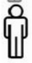 The current generation

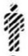 The next generation

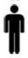 Subsequent generations after the next generations

Table 3: Detailed descriptions with human-shaped icon displays in history from 22 to 35 situations within the 35 ones as seen by each subject (continuum from where we left off in table 2)

Situations	Human-shaped icons in history	% of option A in history	X	D	X/D
22	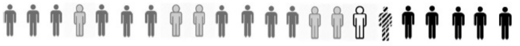	0.67	2600	100	26
23	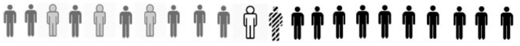	0.70	1500	300	5
24	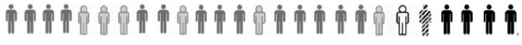	0.70	2200	100	22
25	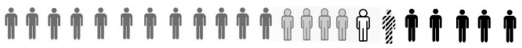	0.75	0	300	0
26	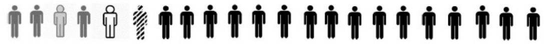	0.75	900	900	1
27	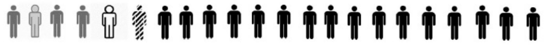	0.75	1800	600	3
28	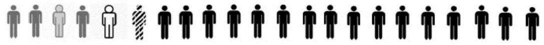	0.75	3300	100	33
29	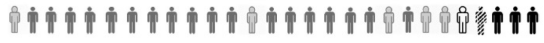	0.78	0	200	0
30	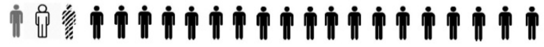	1	1800	1800	1
31	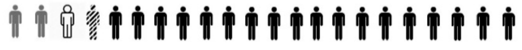	1	1800	900	2
32	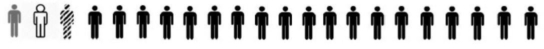	1	2400	1200	2
33	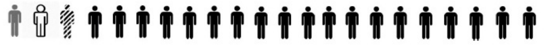	1	3300	300	11
34	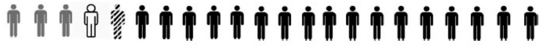	1	3000	200	15
35	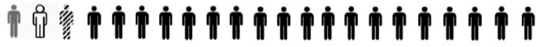	1	3500	100	35

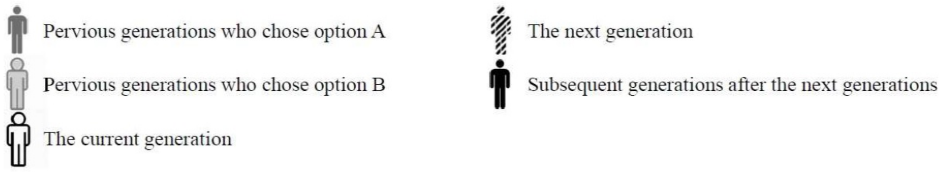

List of Figures

1 Scatter plot for the distribution of the 35 situations in our game 16
2 The 23rd situation of the one-person ISDG as seen by each subject 17
3 Procedures of the one-person ISDG, SVO game and questionnaire in one session . 18
4 The screen of the ISDGs as seen by each subject in chronological order 19

Figure 1: Scatter plot for the distribution of the 35 situations in our game

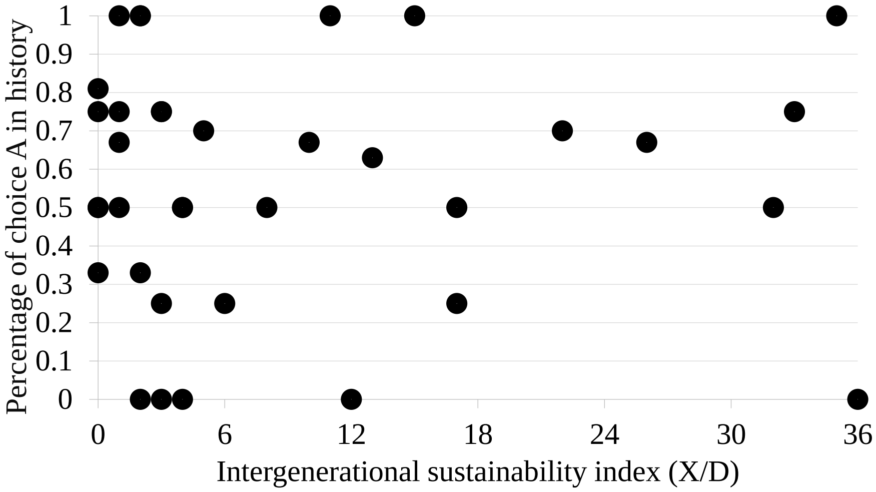

Figure 2: The 23rd situation of the one-person ISDG as seen by each subject

(a) The first screen

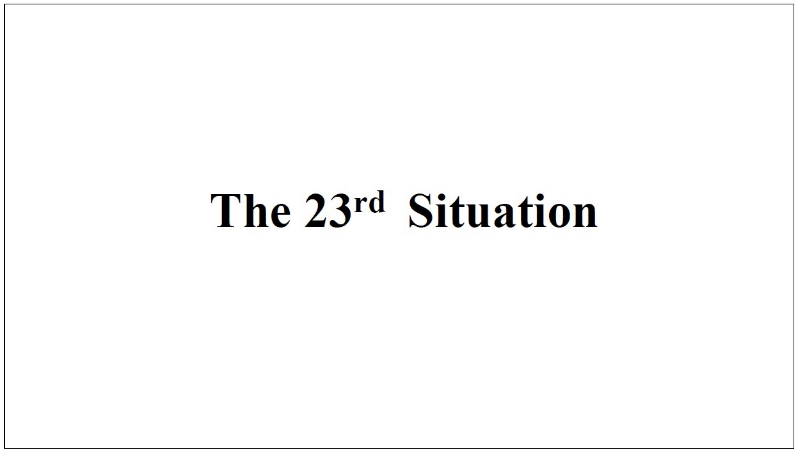

(b) The second screen

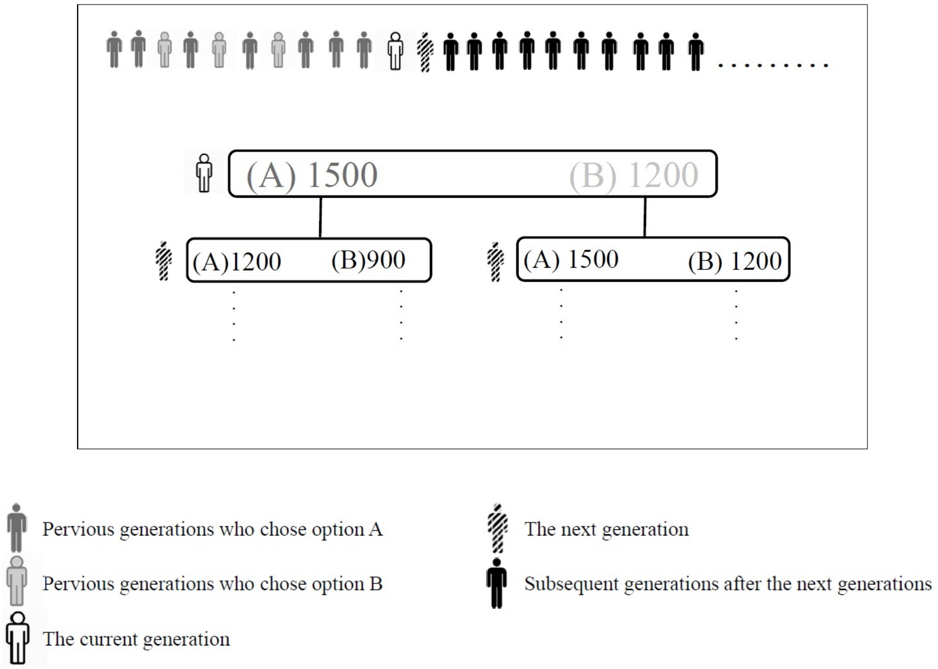

Figure 3: Procedures of the one-person ISDG, SVO game and questionnaire in one session

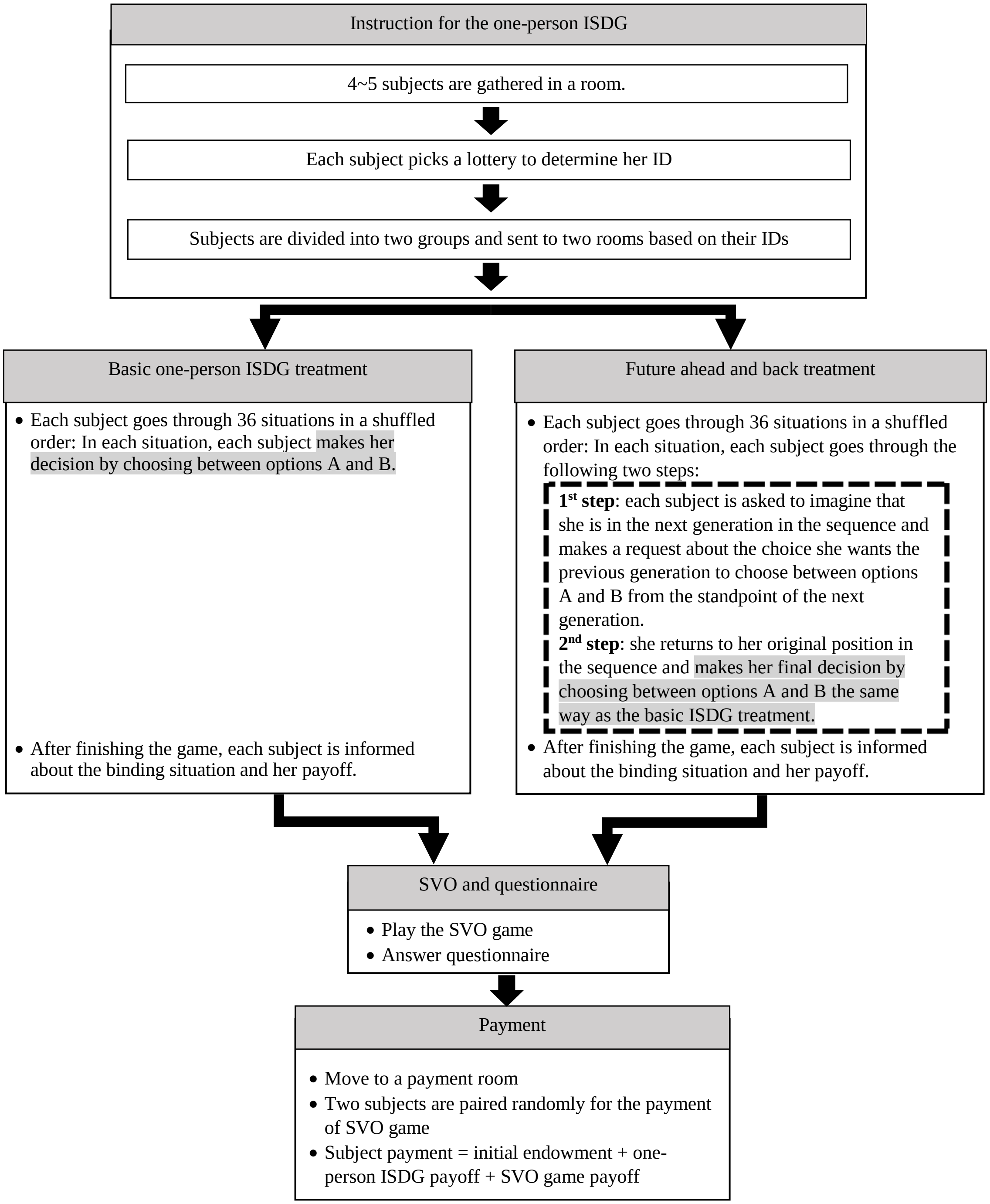

Figure 4: The screen of the ISDGs as seen by each subject in chronological order

(a) One-person ISDG situation for the basic ISDG treatment

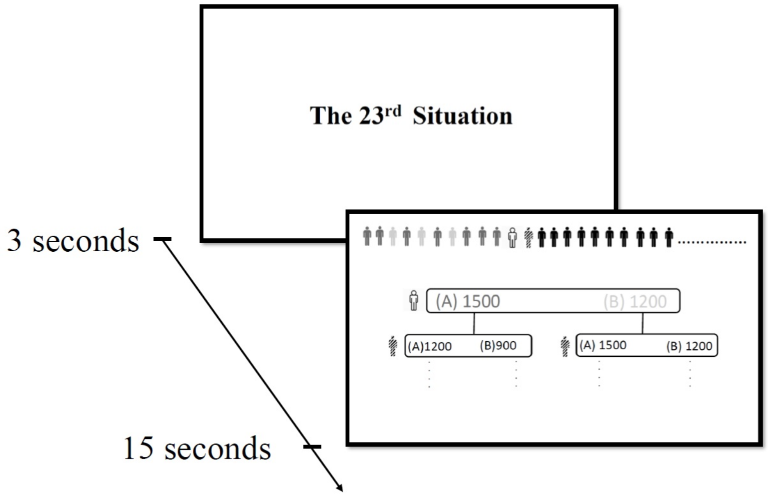

(b) One-person ISDG situation for the FAB treatment

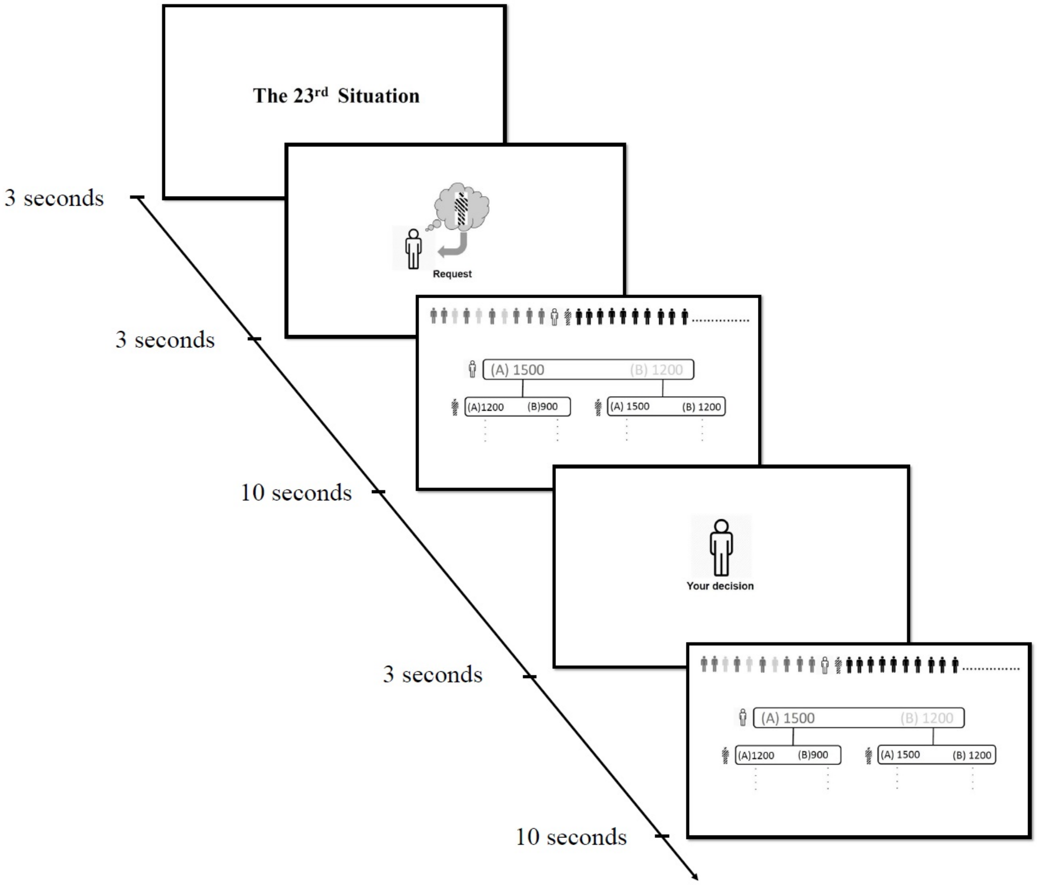

Supplement: Supplementary file 1 — Supplementary material 1 [file 41598_2021_81835_MOESM1_ESM.pdf]
