# Supplementary material for: Intergenerational sustainability is enhanced by taking the perspective of future generations
Source: Sci Rep. 2021 Jan 28;11:2437. doi: 10.1038/s41598-021-81835-y (PMC7844004; doi:10.1038/s41598-021-81835-y)
Supplement: Supplementary file 3 — Supplementary material 3 [file 41598_2021_81835_MOESM3_ESM.pdf]

# Instructions

KUT future design research group

July 24, 2020

## Overview

Thank you for your participation. In this experiment, you will be paid 4000 yen on an average, and each of you shall be given **500 points** as an **initial endowment** only for participation. **Additional points** will be given, depending on how you perform in the experiment that follows. The total payoff you will receive from the experiment is expressed as follows:

$$\textbf{Total payoff} = \textbf{Initial endowment of } 500 \textbf{ points} + 1.5 \times \textbf{Additional points} + \textbf{Bonus points}.$$

From now on, you will go through the following procedures.

1. $AB$ game (**Additional points**),
2. questionnaires and interviews (**Bonus points**).

We will explain each by each from now.

## 1 $AB$ game

You are asked to choose between option $A$ and option $B$ in the "$AB$ **game**."

- By choosing option $A$, you receive $X$
- By choosing option $B$, you receive $X - D$

as the "**additional points**," respectively, where $D$ is a point difference between option $A$ and option $B$. Suppose that $X = 3600$ and $D = 900$. In this case, you receives $3600$ (2700) as the additional points by choosing option $A$ ($B$).

### 1.1 What you choose would affect others in the $AB$ game

What you choose will affect the payoffs for others in the $AB$ game. In the $AB$ game, you are part of a sequence of people consisting of the $1st, 2nd, 3rd, \ldots$ individuals. At the beginning, the $1st$ individual in the sequence plays an $AB$ game with specific values of $X$ and $D$. Given the $1st$ individual's choice between option $A$ and option $B$, the $2nd$ individual plays the $AB$ game as described in table 1. Table 1 shows that the additional points for the $2nd$ individual decrease uniformly by $D$, when the $1st$ individual chooses option $A$. When the $1st$ individual chooses option $B$, the $2nd$ individual can have the same decision environment as the the $1st$ individual faced.

This rule could be better explained and understood with numerical examples.

Table 1: The decision made by the $1st$ individual affects the $2nd$ individual

| $1st$ individual | $2nd$ individual |
|---|---|
| $A$: $X$ | $A$: $X - D$<br>$B$: $X - 2D$ |
| $B$: $X - D$ | $A$: $X$<br>$B$: $X - D$ |

Table 2: A case with $X = 3600$ and $D = 900$: The decision made by the $1st$ individual affects the $2nd$ individual

| $1st$ individual | $2nd$ individual |
|---|---|
| $A$: 3600 | $A$: 2700<br>$B$: 1800 |
| $B$: 2700 | $A$: 3600<br>$B$: 2700 |

**Example 1 (A case when $X = 3600$ and $D = 900$)**
*In this case, table 1 can be reexpressed as table 2.*

- *Additional points for the $1st$ individual:*
  - *When the $1st$ individual in the sequence chooses option $A$, she receives $X = 3600$ points.*
  - *When the $1st$ individual chooses option $B$, she receives $X - D = 3600 - 900 = 2700$ points.*
- *Additional points for the $2nd$ individual:*
  - *When the $1st$ individual chooses option $A$, the additional points the 2nd individual in the sequence can receive by choosing option $A$ and option $B$ uniformly decline by $D = 900$ points and they are 2700 and 1800, respectively (table 2).*
  - *When the $1st$ individual chooses option $B$, the additional points the 2nd individual can receive by choosing option $A$ and option $B$ remain the same, and they are 3600 points and 2700 points, respectively (table 2).* ■

The rule with the same value of $D$ applies to any pair within a single sequence of individuals, say, between the $2nd$ and the $3rd$, between the $4th$ and the $5th$ individuals and so on. To further clarify the rule, another example is presented below.

Table 3: A case with $X = 2700$ and $D = 900$: The decision made by the $2nd$ individual affects the $3rd$ individual, assuming that the $1st$ individual chooses option $A$

| $2nd$ individual | $3rd$ individual |
|---|---|
| $A$: 2700 | $A$: 1800<br>$B$: 900 |
| $B$: 1800 | $A$: 2700<br>$B$: 1800 |

**Example 2 (A case between $2nd$ and $3rd$ individuals as a continuation of example 1)**

*Assume that the $1st$ individual chooses option A in example 1. In this case, the $2nd$ individual will face the $AB$ game where she receives 2700 points or 1800 points by choosing option A and option B, respectively, given the $1st$ individual's choice of option A (see tables 2 and 3). More specifically, the rule with $D = 900$ as described in tables 1 and 2 applies between the $2nd$ and the $3rd$ individuals as in table 3.*

- *When the $2nd$ individual chooses option A, the additional points the 3rd individual can receive by choosing A and B uniformly decline by 900 points, and they are 1800 points and 900 points, respectively (table 3).*
- *When the $2nd$ individual chooses option B, the additional points the $3rd$ individual can receive by choosing A and B remain the same, and they are 2700 points and 1800 points, respectively (table 3).* ■

As illustrated in examples 1 and 2, one individual decision to choose option $A$ uniformly decreases the payoffs the next and subsequent individuals in the sequence can receive by $D = 900$.

**Remark 1.1 (The "$D$" rule)**

*The D is considered*

1. *your point difference between option A and option B, and*
2. *a decline of additional points for the next and subsequent individuals within a single sequence when you choose option A.* ■

Under the "$D$" rule, if more individuals in the sequence choose option $A$, the payoff $X$ associated with option $A$ keeps declining as the sequence progresses and the payoff $X$ associated with option $A$ may become even "negative." Consider a sequence of individuals with $D = 900$, starting $X = 3600$ with the $1st$ individual. In this case, the payoff $X$ associated with option $A$ becomes negative $-900$, when five individuals choose option $A$ in a sequence. In this experiment, each of you will be randomly assigned to be $n$th individual in the sequence and will be asked to decide between option $A$ and option $B$, given the history of previous individuals' decisions as information available to you.

Figure 1: A computer screen for the decision between option $A$ and option $B$

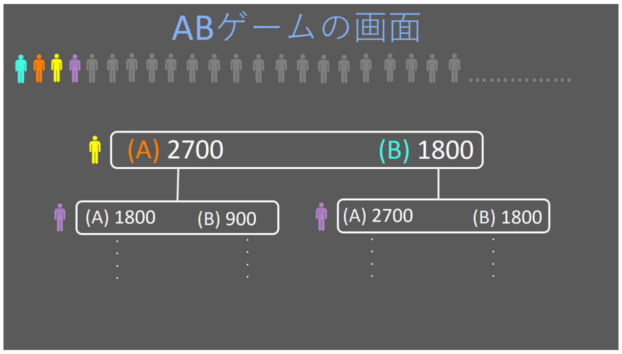


Each of you sees the computer screen that displays the information about previous individuals' choices in a sequence and the "$D$" rule as shown in figure 1, and is asked to make a decision between option $A$ and option $B$. At the top of the screen, there is a queue of human symbols with colors that represent a history of how previous individual chose in the sequence you belong to. The yellow human symbol represents the current individual (you), while the orange (green) human symbol represents the previous individual who has already chosen option $A$ ($B$). The purple human symbol represents the next individual in the sequence after you. The choices in a bigger font size represent the current individual's (your) payoffs options associated with the two options of $A = 2700$ and $B = 1800$, respectively. On the other hand, the next individual's payoffs associated with option $A$ and option $B$ are displayed in a smaller font size, depending on how the current individual (you) chooses.

## A case in another sequence

The value of $D$ between option $A$ and option $B$ may change, sequence by sequence, in the $AB$ game that you will play, while every sequence starts with $X = 3600$ for the $1st$ individual. To illustrate that, another example is provided below.

**Example 3 (A case when D $=$ 300)**

*Consider a sequence of individuals where $D = 300$ and $X$ starts with $3600$ for the $1st$ individual. Suppose $1st, 2nd, 3rd, 4th$ individuals in the sequence chose options $B, A, B, A$, respectively, and you are the $5th$ individual. In this case, two individuals in the sequence chose option A, and therefore, you face the payoffs of option $A = 3000 = 3600 - 300 - 300$ and option $B = 2700 = 3000 - 300$ as shown in table 4, and are asked to make a decision between option $A$ and option $B$ with the computer screen as shown in figure 2. Note that, in this case,*

- *when you choose option $A$, the additional points the $6th$ individual in the sequence can receive by choosing option $A$ and option $B$ uniformly decline by $D = 300$ points, and they are $2700$ and $2400$, respectively (table 4).*
- *when you choose option $B$, the additional points the $6th$ individual in the sequence can receive by choosing option $A$ and option $B$ remain the same, and which are $3000$ points and $2700$ points, respectively (table 4).*

Figure 2: A computer screen for the decision between option $A$ and option $B$ when $D = 300$

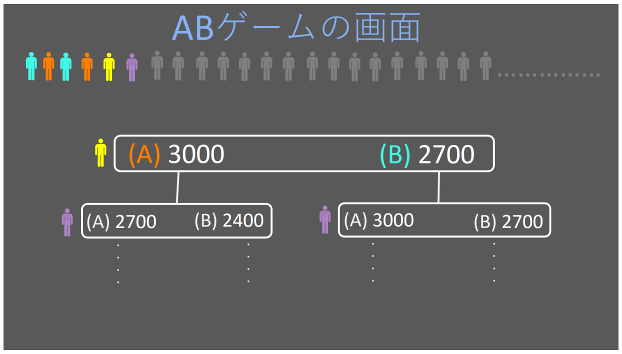


Table 4: How the decision of the $5th$ individual affects the $6th$ individual and $D = 300$

| $5th$ individual | $6th$ individual |
|---|---|
| $A$: 3000 | $A$: 2700<br>$B$: 2400 |
| $B$: 2700 | $A$: 3000<br>$B$: 2700 |

## Summary

The choice of option $A$ or option $B$ made by you affects not only yourself but also all of other subsequent individuals within a single sequence. Note that every sequence starts with $X = 3600$ for the $1st$ individual and the "$D$" rule summarized in remark 1.1 in the $AB$ game apply to any pair of individuals in a sequence between $3rd$ and $4th$ and between $4th$ and $5th$ individuals, . . . and so on.

## A whole procedure for the $AB$ game

You will be randomly assigned to be part of a sequence of individuals and be asked to decide between option $A$ and option $B$ given previous individuals' choices. In total, you will experience being part of 36 different sequences of individuals where the value of $D$ may change, sequence by sequence. In other words, you are asked to decide between option $A$ and option $B$ 36 times by being part of 36 different sequences. For example, you may be asked to decide between option $A$ and option $B$ as the $4th$ individual in one sequence with $D = 100$ given the history of previous individuals' choices. In another situation, you may be asked to decide between option $A$ and option $B$ by being the $2nd$ individual in a sequence with $D = 600$, and so on. In total, you are asked to experience and decide in 36 different situations. After you decide in 36 different sequences, one sequence out of 36 sequences you have gone through in the $AB$ game shall be randomly chosen to determine your payoff as your "additional points." Your payoff from the $AB$ game is calculated to be $1.5 \times$ additional points. In other words, one point you get as additional points equals 1.5 Japanese yen.

**Please remember what you will decide in 36 different situations affect not only your payoff but also others' payoffs in a sequence that follow after you.**

**Therefore, please seriously consider which to choose between option $A$ and option $B$ in each of 36 different situations.**
