# Supplementary material for: Intergenerational sustainability is enhanced by taking the perspective of future generations
Source: Sci Rep. 2021 Jan 28;11:2437. doi: 10.1038/s41598-021-81835-y (PMC7844004; doi:10.1038/s41598-021-81835-y)
Supplement: Supplementary file 4 — Supplementary material 4 [file 41598_2021_81835_MOESM4_ESM.pdf]

Instructions

KUT future design research group

July 24, 2020

Overview

Thank you for your participation. In this experiment, you will be paid 4000 yen on an average, and each of you shall be given **500 points** as an **initial endowment** only for participation. **Additional points** will be given, depending on how you perform in the experiment that follows. The total payoff you will receive from the experiment is expressed as follows:

$$\textbf{Total payoff} = \textbf{Initial endowment of 500 points} + 1.5 \times \textbf{Additional points} + \textbf{Bonus points}.$$

From now on, you will go through the following procedures.

1. AB game (**Additional points**),
2. questionnaires and interviews (**Bonus points**).

We will explain each by each from now.

1 AB game

You are asked to choose between option A and option B in the "AB **game**."

- By choosing option A, you receive X
- By choosing option B, you receive $X - D$

as the "**additional points**," respectively, where D is a point difference between option A and option B. Suppose that $X = 3600$ and $D = 900$. In this case, you receives 3600 (2700) as the additional points by choosing option A (B).

1.1 What you choose would affect others in the AB game

What you choose will affect the payoffs for others in the AB game. In the AB game, you are part of a sequence of people consisting of the $1st, 2nd, 3rd, \ldots$ individuals. At the beginning, the $1st$ individual in the sequence plays an AB game with specific values of X and D. Given the $1st$ individual's choice between option A and option B, the $2nd$ individual plays the AB game as described in table 1. Table 1 shows that the additional points for the $2nd$ individual decrease uniformly by D, when the $1st$ individual chooses option A. When the $1st$ individual chooses option B, the $2nd$ individual can have the same decision environment as the the $1st$ individual faced.

This rule could be better explained and understood with numerical examples.

Table 1: The decision made by the $1st$ individual affects the $2nd$ individual

$1st$ individual	$2nd$ individual
A: X	A: $X - D$<br>B: $X - 2D$
B: $X - D$	A: X<br>B: $X - D$

Table 2: A case with $X = 3600$ and $D = 900$: The decision made by the $1st$ individual affects the $2nd$ individual

$1st$ individual	$2nd$ individual
A: 3600	A: 2700<br>B: 1800
B: 2700	A: 3600<br>B: 2700

Example 1 (A case when $X = 3600$ and $D = 900$)

In this case, table 1 can be reexpressed as table 2.

- *Additional points for the $1st$ individual:*
  - *When the $1st$ individual in the sequence chooses option A, she receives $X = 3600$ points.*
  - *When the $1st$ individual chooses option B, she receives $X - D = 3600 - 900 = 2700$ points.*
- *Additional points for the $2nd$ individual:*
  - *When the $1st$ individual chooses option A, the additional points the 2nd individual in the sequence can receive by choosing option A and option B uniformly decline by $D = 900$ points and they are 2700 points and 1800 points, respectively (table 2).*
  - *When the $1st$ individual chooses option B, the additional points the 2nd individual can receive by choosing option A and option B remain the same, and they are 3600 points and 2700 points, respectively (table 2).* ■

The rule with the same value of D applies to any pair within a single sequence of individuals, say, between the $2nd$ and the $3rd$, between the $3rd$ and the $4th$ individuals and so on. To further clarify the rule, another example is presented below.

Example 2 (A case between $2nd$ and $3rd$ individuals as a continuation of example 1)

Table 3: A case with $X = 2700$ and $D = 900$: The decision made by the $2nd$ individual affects the $3rd$ individual, assuming that the $1st$ individual chooses option A

$2nd$ individual	$3rd$ individual
A: 2700	A: 1800<br>B: 900
B: 1800	A: 2700<br>B: 1800

Assume that the $1st$ individual chooses option A in example 1. In this case, the $2nd$ individual will face the AB game where she receives 2700 points or 1800 points by choosing option A and option B, respectively (see tables 2 and 3). More specifically, the rule with $D = 900$ as described in tables 1 and 2 applies between the $2nd$ and the $3rd$ individuals as in table 3.

- *When the $2nd$ individual chooses option A, the additional points the $3rd$ individual can receive by choosing A and B uniformly decline by 900 points, and they are 1800 points and 900 points, respectively (table 3).*
- *When the $2nd$ individual chooses option B, the additional points the $3rd$ individual can receive by choosing A and B remain the same, and they are 2700 points and 1800 points, respectively (table 3).* ■

As illustrated in examples 1 and 2, one individual decision to choose option A uniformly decreases the payoffs the next and subsequent individuals in the sequence can receive by $D = 900$.

Remark 1.1 (The "D" rule)

The D is considered

1. *your point difference between option A and option B, and*
2. *a decline of additional points for the next and subsequent individuals within a single sequence when you choose option A.* ■

Under the "D" rule, if more individuals in the sequence choose option A, the payoff X associated with option A keeps declining as the sequence progresses and may become even "negative." Consider a sequence of individuals with $D = 900$, starting $X = 3600$ for the $1st$ individual. In this case, the payoff X associated with option A becomes negative -900, when five individuals in a sequence choose option A. In this experiment, each of you will be randomly assigned to be nth individual in a sequence and will be asked to decide between option A and option B, given the history of previous individuals' decisions as information available to you.

Each of you sees the computer screen that displays the information about previous individuals' choices in a sequence and the "D" rule as shown in figure 1, and is asked to make a decision between option A and option B. At the top of the screen, there is a queue of human symbols with colors that represent a history of

Figure 1: A computer screen for the decision between option A and option B

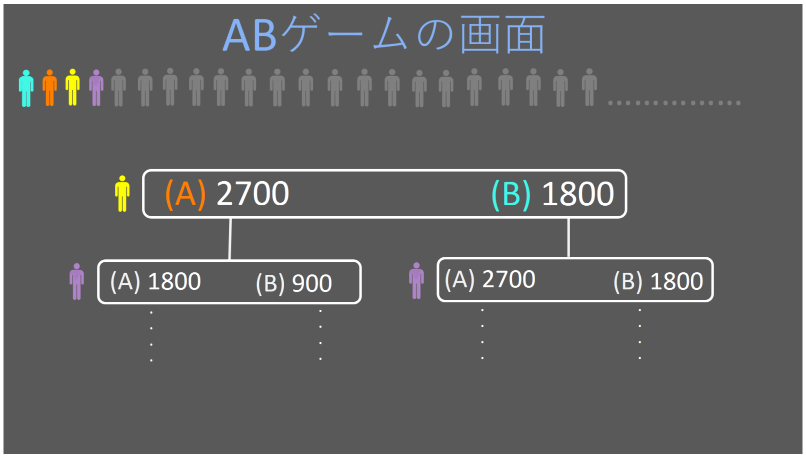

how previous individuals chose in the sequence you belong to. The yellow human symbol represents the current individual (you), while the orange (green) human symbol represents the previous individual who has already chosen option A (B). The purple human symbol represents the next individual in the sequence after you. The choices in a bigger font size represent the current individual's (your) payoffs associated with the two options of $A = 2700$ and $B = 1800$, respectively. On the other hand, the next individual's payoffs associated with option A and option B are displayed in a smaller font size, depending on how the current individual (you) chooses.

A case in another sequence

The value of D between option A and option B may change, sequence by sequence, in the AB game that you will play, while every sequence starts with $X = 3600$ for the $1st$ individual. To illustrate that, another example is provided below.

Example 3 (A case when D $=$ 300)

Consider a sequence of individuals where $D = 300$ and X starts with 3600 for the $1st$ individual. Suppose $1st, 2nd, 3rd, 4th$ individuals in the sequence chose options B, A, B, A, respectively, and you are the $5th$ individual. In this case, two individuals in the sequence chose option A, and therefore, you face the payoffs of option $A = 3000 = 3600 - 300 - 300$ and option $B = 2700 = 3000 - 300$ as shown in table 4, and are asked to make a decision between option A and option B with the computer screen as shown in figure 2. Note that, in this case,

- *when you choose option A, the additional points the $6th$ individual in the sequence can receive by choosing option A and option B uniformly decline by $D = 300$ points, and they are 2700 points and 2400 points, respectively (table 4).*

- *when you choose option B, the additional points the $6th$ individual in the sequence can receive by choosing option A and option B remain the same, and which are 3000 points and 2700 points, respectively (table 4).*

Figure 2: A computer screen for the decision between option A and option B when $D = 300$

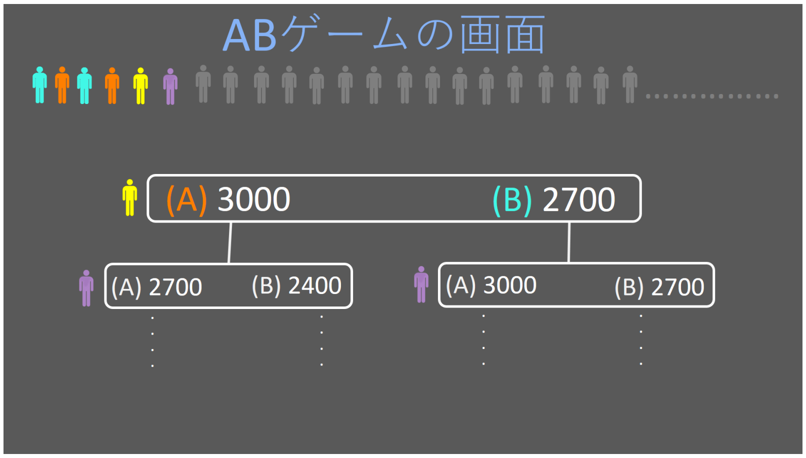

Table 4: How the decision of the $5th$ individual affects the $6th$ individual and $D = 300$

$5th$ individual	$6th$ individual
A: 3000	A: 2700<br>B: 2400
B: 2700	A: 3000<br>B: 2700

Figure 3: A computer screen that is displayed to ask you be in the position of the next individual in a sequence

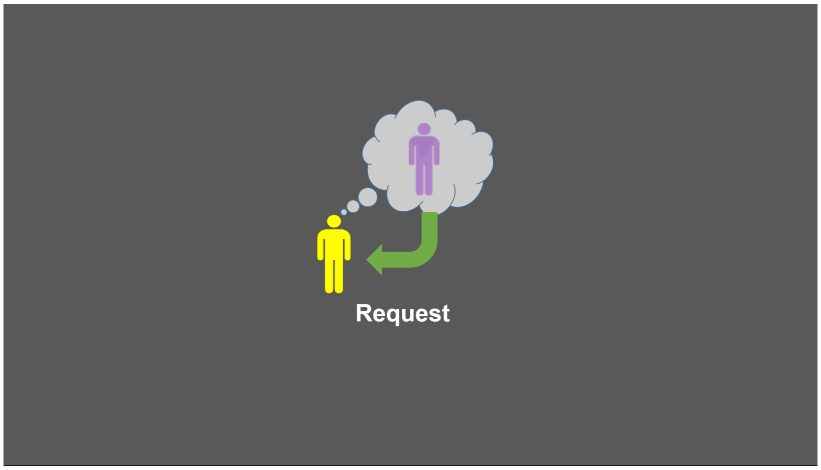

Summary

Your choice between option A and option B affects not only yourself but also all of other subsequent individuals within a single sequence. Note that every sequence starts with $X = 3600$ for the $1st$ individual and the "D" rule summarized in remark 1.1 in the AB game apply to any pair of individuals in a sequence between $3rd$ and $4th$ and between $4th$ and $5th$ individuals, . . . and so on. In choosing between option A and option B, you will be asked to take the following decision making steps:

Remark 1.2 (Decision making steps)

There are three steps to follow.

Step 1: You are first asked to imagine that you are in the position of the next individual in the same sequence. For example, if your position is the ith individual in a sequence, you are first asked to imagine that you are the $i + 1$th individual of that sequence. Next, you are asked to make a request about what you may want the ith individual to choose between option A and option B to the ith individual as if you are the $i + 1$th individual in the sequence.

Step 2: You are asked to be in your original position as the ith individual in the sequence and to choose between option A and option B.

Procedures of decision making steps

The procedures of decision making steps shall be explained by using example 3 in which you are assumed to be the $5th$ individual in a sequence with $D = 300$ and the history in which the $2nd$ and $4th$ individuals chose option A.

Step 1: (a) Figure 2 shall be displayed for 4 seconds to let you understand your decision environment. Next, figure 3 shall be displayed for 3 seconds to ask you to imagine being in the position of the next individual or the $6th$ individual (purple human symbol), and to make a request to the $5th$ individual (yellow human symbol) about what you may want the pervious individual ($5th$ individual) in the sequence to choose between option A and option B based on the additional points for the next individual (the $6th$ individual) as shown in figure 2.

Figure 4: A computer screen that is displayed to ask you to get back to your original position

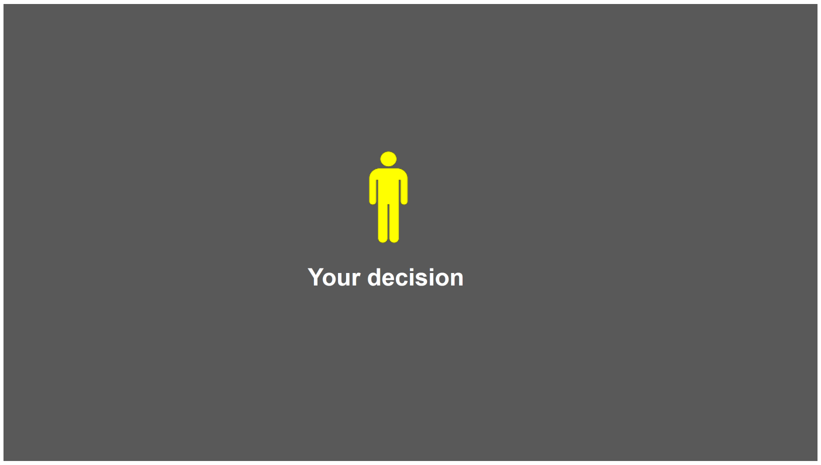

(b) Then, figure 2 shall be redisplayed for 10 seconds for you to make a request to the $5th$ individual (yellow human symbol) from the position of the $6th$ individual (purple human symbol) in the sequence about choosing between option A and option B.

Step 2: (a) Next, figure 4 shall be displayed for 3 seconds to ask you to get back to your original position as the $5th$ individual (yellow human symbol) in the sequence.

(b) Lastly, figure 2 shall be redisplayed for 10 seconds for you to decide between option A and option B as the $5th$ individual in the sequence.

A whole procedure for the AB game

Following a certain rule we set, you will be assigned to be part of a sequence of individuals and be asked to decide between option A and option B given previous individuals' choices and following "procedures of decision making steps." You will experience being part of 36 different sequences of individuals where the value of D may change, sequence by sequence. In other words, you are asked to decide between option A and option B 36 times by being part of 36 different sequences. For example, you may be asked to decide between option A and option B as the $4th$ individual in one sequence with $D = 100$ given the history of previous individuals' choices. In another situation, you may be asked to decide between option A and option B by being the $2nd$ individual in a sequence with $D = 600$, and so on. In total, you are asked to experience and decide in 36 different situations. After you decide in 36 different sequences, one sequence out of 36 sequences you have gone through in the AB game shall be chosen to determine your payoff as your "additional points," following a certain rule we set. Your payoff from the AB game is calculated to be $1.5 \times$ additional points. In other words, one point you get as additional points equals 1.5 Japanese yen.

Please remember that (i) which sequence to be chosen to determine your payoff is NOT known to you "in advance" and that (ii) what you will decide in 36 different situations could affect not only your payoff but also others' payoffs in a sequence that follow after you. Therefore, please seriously consider which to choose between option A and option B in each of 36 different situations.
